# Transgelin 2 guards T cell lipid metabolic programming and anti-tumor function

**DOI:** 10.21203/rs.3.rs-3683989/v1

**Published:** 2023-12-14

**Authors:** Sung-Min Hwang, Deepika Awasthi, Jieun Jeong, Tito A. Sandoval, Chang-Suk Chae, Yusibeska Ramos, Chen Tan, Matías Marin Falco, Ian T. McBain, Bikash Mishra, Lionel B. Ivashkiv, Dmitriy Zamarin, Evelyn Cantillo, Eloise Chapman-Davis, Kevin Holcomb, Diana K. Morales, Paulo C. Rodriguez, Jose R. Conejo-Garcia, Martin Kaczocha, Anna Vähärautio, Minkyung Song, Juan R. Cubillos-Ruiz

**Affiliations:** 1 Department of Obstetrics and Gynecology, Weill Cornell Medicine. New York, NY 10065, USA.; 2 Sandra and Edward Meyer Cancer Center, Weill Cornell Medicine. New York, NY 10065, USA.; 3 Cancer Biology and Genetics Program, Memorial Sloan-Kettering Cancer Center, New York, NY 10065, USA.; 4 Research Program in Systems Oncology, Research Programs Unit, Faculty of Medicine, University of Helsinki, Helsinki, Finland.; 5 Weill Cornell Graduate School of Medical Sciences. New York, NY 10065. USA.; 6 HSS Research Institute and David Z. Rosensweig Genomics Research Center, Hospital for Special Surgery, New York, NY, USA.; 7 Tisch Cancer Institute, Icahn School of Medicine at Mount Sinai, New York, NY 10029, USA.; 8 Department of Immunology, H. Lee Moffitt Cancer Center & Research Institute. Tampa, FL, USA.; 9 Department of Integrated Immunobiology, Duke School of Medicine, Durham, NC 27710, USA.; 10 Duke Cancer Institute, Duke School of Medicine, Durham, NC 27710, USA.; 11 Department of Anesthesiology, Renaissance School of Medicine, Stony Brook University, Stony Brook, NY, USA.; 12 Institute of Chemical Biology and Drug Discovery, Stony Brook University, Stony Brook, NY, USA.; 13 Stony Brook University Pain and Analgesia Research Center (SPARC), Renaissance School of Medicine, Stony Brook University, Stony Brook, NY, USA.; 14 Foundation for the Finnish Cancer Institute, Helsinki, Finland.

## Abstract

Mounting effective immunity against pathogens and tumors relies on the successful metabolic programming of T cells by extracellular fatty acids^1–3^. During this process, fatty-acid-binding protein 5 (FABP5) imports lipids that fuel mitochondrial respiration and sustain the bioenergetic requirements of protective CD8^+^ T cells^4,5^. Importantly, however, the mechanisms governing this crucial immunometabolic axis remain unexplored. Here we report that the cytoskeletal organizer Transgelin 2 (TAGLN2) is necessary for optimal CD8^+^ T cell fatty acid uptake, mitochondrial respiration, and anti-cancer function. We found that TAGLN2 interacts with FABP5, enabling the surface localization of this lipid importer on activated CD8^+^ T cells. Analysis of ovarian cancer specimens revealed that endoplasmic reticulum (ER) stress responses elicited by the tumor microenvironment repress TAGLN2 in infiltrating CD8^+^ T cells, enforcing their dysfunctional state. Restoring TAGLN2 expression in ER-stressed CD8^+^ T cells bolstered their lipid uptake, mitochondrial respiration, and cytotoxic capacity. Accordingly, chimeric antigen receptor T cells overexpressing TAGLN2 bypassed the detrimental effects of tumor-induced ER stress and demonstrated superior therapeutic efficacy in mice with metastatic ovarian cancer. Our study unveils the role of cytoskeletal TAGLN2 in T cell lipid metabolism and highlights the potential to enhance cellular immunotherapy in solid malignancies by preserving the TAGLN2-FABP5 axis.

T cell function and fate are dictated by nutrient availability and the precise regulation of multiple metabolic pathways ^6–8^. For instance, the efficient import, trafficking, and catabolism of extracellular fatty acids is crucial to fulfill the bioenergetic demands of effector and memory CD8^+^ T cells^2,3,9,10^. The fatty acid binding protein 5 (FABP5) plays a pivotal role in this process as a dominant transporter of extracellular fatty acids that mitochondria utilize for energy production through fatty acid β-oxidation (FAO)^11,12^. While the FABP5-FAO axis has been demonstrated to be critical for the development and maintenance of adaptive immunity to pathogens and tumors^4,5,13^, the mechanisms regulating this major immunometabolic pathway remain largely unexplored.

Metastatic ovarian cancer (OvCa) is a prototypical immunosuppressive malignancy, known for its resistance to standard treatments and all forms of T cell-based immunotherapy^14–16^. This daunting clinical scenario indicates that unconventional, yet to be identified, mechanisms of T cell suppression remain actively engaged in the OvCa environment despite major treatment efforts. Indeed, T cells infiltrating ovarian tumors are normally retained in a dysfunctional state characterized by bioenergetic anomalies, aberrant activation of cellular stress responses, and negligible effector function that cannot be reversed through classical immunotherapeutic approaches^17–19^. Whether aggressive epithelial malignancies, such as OvCa, disrupt FABP5-mediated lipid metabolic programming in T cells to evade adaptive immune control is unknown.

Here, we uncover that the cytoskeletal protein TAGLN2 cooperates with FABP5 to enable efficient fatty acid import and utilization by CD8^+^ T cells. TAGLN2 is silenced in dysfunctional intratumoral CD8^+^ T cells by intrinsic endoplasmic reticulum (ER) stress responses. Preserving TAGLN2 expression enhances the lipid uptake, bioenergetic, and functional capacities of ER-stressed T cells and thus improves the effectiveness of adoptive cellular immunotherapy in mice with metastatic OvCa.

## RESULTS

### OvCa impairs lipid uptake and FABP5 surface localization in CD8^+^ T cells.

**Figure 1.**
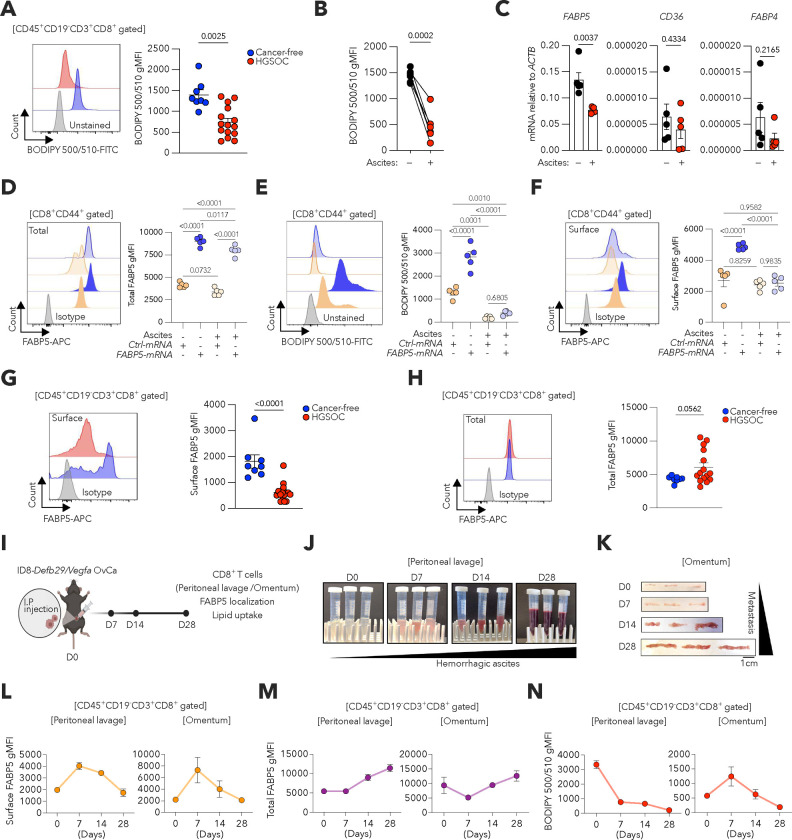
Defective lipid uptake and FABP5 surface localization in OvCa-infiltrating CD8^+^ T cells. **a**, Representative FACS histograms and quantitative analysis of lipid uptake (BODIPY 500/510) for CD45^+^CD19^−^CD3^+^CD8^+^ T cells isolated from peripheral blood of cancer-free women (*n* = 8) or malignant ascites of HGSOC patients (*n* = 15). **b,c**, Human naïve CD8^+^ T cells from peripheral blood of cancer-free women were activated via CD3/CD28 stimulation for 32 h, followed by 16 h in the absence or presence of 50% HGSOC ascites supernatants. (**b**) Lipid uptake was assessed by FACS (*n* = 5). (**c**) *FABP5, CD36* and *FABP4* expression was determined via qRT-PCR and data were normalized to endogenous levels of *ACTB* (*n* = 5). **d-f**, Human naïve CD8^+^ T cells from peripheral blood of cancer-free individuals were activated via CD3/CD28 stimulation and then Neon-electroporated with either control (*Ctrl*) or human *FABP5* mRNAs. Cells were expanded and treated with 0% or 50% of HGSOC ascites supernatants. Representative histograms and quantitative analysis of total FABP5 protein levels (**d**), lipid uptake (**e**), and cell surface FABP5 protein levels (**f**) in CD8^+^CD44^+^ T cells determined by FACS (*n* = 5 per group). **g,h**, Representative FACS histograms and quantitative analysis of cell surface (**g**) and total (**h**) FABP5 protein levels in CD45^+^CD19^−^CD3^+^CD8^+^ T cells isolated from the same specimens described in **a**. **i-n**, C57BL/6J female mice (*n* = 9) were intraperitoneally injected with ID8-*Defb29/Vegfa* OvCa cells and euthanized on days 7, 14, or 28 after tumor implantation (*n* = 3 per group). Experimental scheme and readouts (**i**). Representative images of peritoneal lavage (**j**) and omentum (**k**) from each group. FACS-based analysis to determine the kinetics of cell surface FABP5 protein levels (**l**), total FABP5 protein levels (**m**), and lipid uptake (**n**) in CD45^+^CD19^−^CD3^+^CD8^+^ T cells from peritoneal lavage or omentum (*n* = 3 per group). Data are presented as mean ± s.e.m. **a,c,g,h**, Two-tailed unpaired Student’s t-test. **b**, Two-tailed paired Student’s t-test. **d-f**, One-way ANOVA with Tukey’s multiple comparisons test. Exact *P-values* are shown. The ‘*n*’ values represent biologically independent samples. gMFI, Geometric mean fluorescence intensity.

We sought to determine whether OvCa-infiltrating CD8^+^ T cells exhibit altered capacity to import extracellular fatty acids. FACS-based analyses using the fluorescent fatty acid analog C1-BODIPY 500/510 C12 showed that CD8^+^ T cells residing in the ascites of patients with high-grade serous OvCa (HGSOC), the most common and aggressive form of OvCa^20^, demonstrate defective lipid uptake compared with peripheral CD8^+^ T cells isolated from cancer-free women (Fig. 1a). Of note, in vitro exposure to HGSOC ascites supernatants markedly impaired extracellular fatty acid uptake by activated CD8^+^ T cells isolated from cancer-free donors (Fig. 1b). This defect was accompanied by a ~50% reduction in *FABP5* expression, but not in genes encoding other lipid transporters such as CD36 and FABP4 (Fig. 1c). We therefore tested whether increasing *FABP5* transcript levels could rescue this process. Intriguingly, *FABP5* overexpression via transient mRNA electroporation augmented the total protein levels of this lipid transporter in activated CD8^+^ T cells exposed to HGSOC ascites supernatants (Fig. 1d) but failed to restore their fatty acid uptake capacity (Fig. 1e). Since FABP5 can be found in the cytosol or on the plasma membrane^21^, these results prompted us to investigate whether OvCa might alter the localization and/or activity of FABP5 to restrain extracellular lipid transport into CD8^+^ T cells.

**Extended Data Figure 1.**
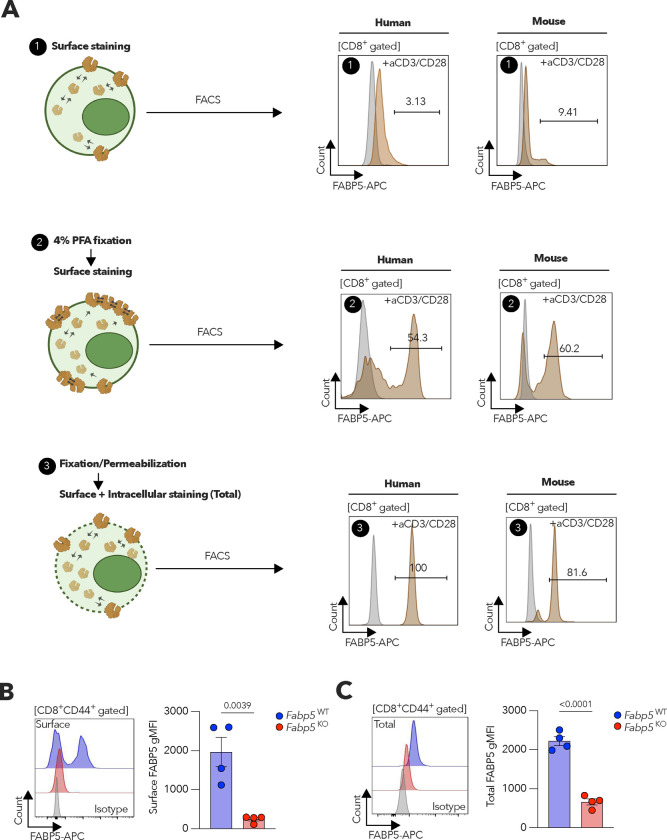
Assessment of FABP5 localization in activated human and mouse CD8^+^ T cells. **a**, Scheme illustrating the different strategies to detect cell surface or total (surface + intracellular) FABP5 protein expression in activated CD8^+^ T cells from both human and mouse. Representative FACS histograms depict FABP5 staining, with gray peaks representing isotype controls. **b,c**, Representative histograms and quantitative analysis of cell surface (**b**) and total (**c**) levels of FABP5 protein expression determined by FACS in gated CD8^+^CD44^+^ T cells from WT or *Fabp5* knockout (KO) mice (*n* = 4 per genotype). Data are presented as mean ± s.e.m. **b,c**, Two-tailed unpaired Student’s t-test. *P*<0.05 is considered to be statistically significant and exact *P-values* are shown. The ‘*n*’ values represent biologically independent samples. gMFI, Geometric mean fluorescence intensity.

We optimized a FACS-based immunofluorescence staining method to discern the expression levels of surface-localized versus total FABP5 in human and mouse CD8^+^ T cells (Extended Data Fig. 1a), which was validated using *Fabp5* knockout mice (Extended Data Fig. 1b,c). *FABP5* mRNA supplementation significantly increased surface FABP5 expression when human CD8^+^ T cells were kept in normal culture media, but not upon exposure to HGSOC patient-derived ascites (Fig. 1f). Consistent with these in vitro findings, CD8^+^ T cells isolated from the ascites of HGSOC patients demonstrated a significant reduction in surface FABP5 expression compared with peripheral CD8^+^ T cells from cancer-free women (Fig. 1g), although their total FABP5 levels were comparable (Fig. 1h).

We next evaluated the status of surface FABP5 expression and lipid uptake in tumor-associated CD8^+^ T cells throughout the development of metastatic OvCa. To this end, we used the orthotopic ID8-*Defb29*/*Vegfa* mouse model^22^ (Fig. 1i), which progressively generates an immunosuppressive peritoneal carcinomatosis characterized by ascites accumulation and omental metastases (Fig. 1j,k) that recapitulate the advanced stages of human OvCa^23–27^. Notably, surface FABP5 expression increased on CD8^+^ T cells in the peritoneal cavity and omentum during the first week of tumor progression, compared with their counterparts in naïve mice, but markedly declined thereafter as ascites and omental metastatic lesions developed (Fig. 1l). In sharp contrast, total FABP5 expression in the same CD8^+^ T cells analyzed increased throughout tumor progression (Fig. 1m). Consistent with the observed reduction in surface FABP5 expression, CD8^+^ T cells infiltrating these tumor locations also demonstrated major defects in fatty acid uptake over time (Fig. 1n). These results uncover that the ovarian tumor microenvironment disrupts the surface localization of FABP5 in CD8^+^ T cells, limiting their ability to import extracellular fatty acids.

### TAGLN2 enables FABP5-driven lipid uptake in CD8^+^ T cells.

**Extended Data Figure 2.**
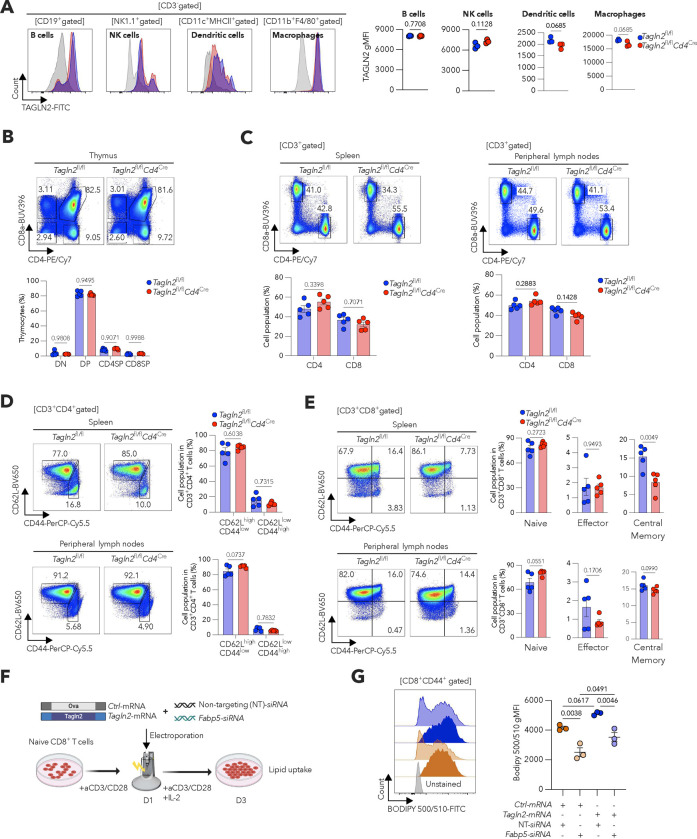
Characterization of mice lacking TAGLN2 in T cells and analysis of lipid uptake in *Fabp5*-silenced CD8 T cells overexpressing *Tagln2*. **a**, Representative histograms and quantitative analysis of TAGLN2 protein expression in B cells (CD3^−^CD19^+^), natural killer (NK) cells (CD3^−^NK1.1^+^), dendritic cells (CD3^−^CD11c^+^MHCll^+^) and macrophages (CD3^−^CD11b^+^F4/80^+^) in the spleen and peripheral lymph nodes of *Tagln2*^f/f^ or *Tagln2*^f/f^ *Cd4*^cre^ mice (*n* = 3 per genotype). **b**, Representative FACS plots and quantitative analysis of double negative (CD4^−^CD8^−^), double positive (CD4^+^CD8^+^), or single positive (CD4^+^ or CD8^+^) thymocytes frequencies in the thymus (*n* = 5 per genotype). **c**, Representative FACS plots and quantitative analysis of CD3^+^CD4^+^ or CD3^+^CD8^+^ T cell frequencies in the spleen (left) and peripheral lymph nodes (right) (*n* = 5 per genotype). **d,e**, Expression of CD44 and CD62L on CD3^+^CD4^+^ (**d**) and CD3^+^CD8^+^ (**e**) T cells in the spleen (top) and peripheral lymph nodes (bottom) (*n* = 5 per group). Representative FACS plots and quantitative analysis of the indicated cell populations. Naïve (CD62L^high^CD44^low^), effector (CD62L^low^CD44^high^) and Central memory (CD62L^high^CD44^high^). **f,g**, Naïve CD8^+^ T cells from WT C57BL/6J mice were activated via CD3/CD28 stimulation for 24 h and then Neon-electroporated with either control (*Ctrl*) or mouse *Tagln2* mRNAs, along with non-targeting (control) or *Fabp5*-specific siRNAs. Experimental scheme and readouts (**f**). Representative histograms and quantitative analysis of lipid uptake in CD8^+^CD44^+^ T cells determined by FACS (*n* = 3 per condition) (**g**). Data are presented as mean ± s.e.m. **a-e**, Two-tailed unpaired Student’s t-test. **g**, One-way ANOVA. *P*<0.05 is considered statistically significant and exact *P-values* are shown. The ‘*n*’ values represent biologically independent samples. gMFI, Geometric mean fluorescence intensity.

**Figure 2.**
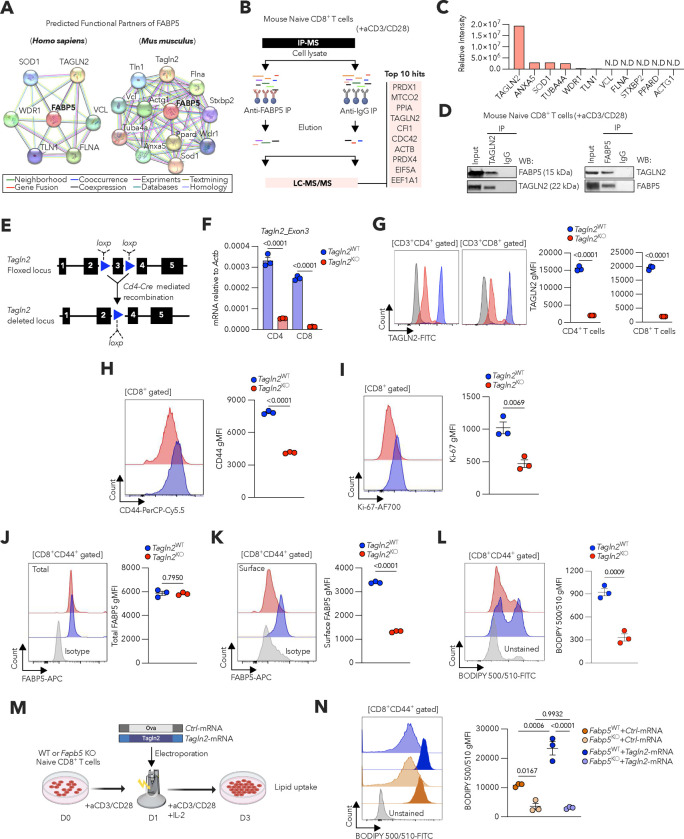
TAGLN2 is required for FABP5 surface localization and lipid uptake in activated CD8^+^ T cells. **a**, Protein-protein interaction maps generated by the STRING database using FABP5 as query in human (*Homo sapiens*) and mouse (*Mus musculus*). **b,c**, Schematic of the immunoprecipitation coupled to mass spectrometry (IP-MS) approach to identify FABP5-interacting proteins. A total of 2,455 proteins were identified at a 1% FDR. Relative intensity was used to compare protein abundance across samples. The top 10 proteins that significantly interact with FABP5 are listed (**b**). The abundance of predicted FABP5-interacting proteins from (**a**) is listed by intensity (**c**). N.D, not detected. **d**, Interaction between FABP5 and TAGLN2 assessed by co-immunoprecipitation using activated CD8^+^ T cells from C57BL/6J mice. Representative image from three independent experiments is shown. **e**, Description of the *Talgln2* deletion strategy depicting floxed and deleted alleles. **f,g**, Deletion efficiency was analyzed in activated CD4^+^ or CD8^+^ T cells from *Tagln2*^fl/fl^ or *Tagln2*^fl/fl^*Cd4*^Cre^ mice via qRT-PCR using a primer set that specifically detects the exon 3 region of *Tagln2*. Data were normalized to *Actb* (**f**). The intracellular levels of TAGLN2 protein were evaluated by FACS (**g**) (*n* = 3 per genotype). **h-l**, WT or TAGLN2-deficient naïve CD8^+^ T cells isolated from the spleen and lymph nodes were activated via CD3/CD28 stimulation for 24 h. Representative histograms and quantitative analysis of CD44 (**h**) and Ki-67 (**i**) expression are shown. Total FABP5 (**j**), surface FABP5 (**k**), and lipid uptake (**l**) by CD8^+^CD44^+^ T cells of the indicated genotypes are shown (*n* = 3 per genotype). **m,n**, Wild-type (WT) or FABP5-deficient naïve CD8^+^ T cells from the spleen and lymph nodes were activated via CD3/CD28 stimulation for 24 h and then Neon-electroporated with either control (*Ctrl*) or mouse *Tagln2* mRNAs. Experimental scheme and readouts (**m**). Representative histograms and quantitative analysis of lipid uptake (BODIPY 500/510) in CD8^+^CD44^+^ T cells determined by FACS (*n* = 3 per condition) (**n**). Data are presented as mean ± s.e.m. **f-l**, Two-tailed unpaired Student’s t-test. **n**, One-way ANOVA with Tukey’s multiple comparisons test. Exact *P-values* are shown. The ‘*n*’ values represent biologically independent samples. gMFI, Geometric mean fluorescence intensity.

We sought to define the molecular mechanisms controlling FABP5 trafficking to the plasma membrane in activated CD8^+^ T cells. STRING database^28^ analyses suggested that FABP5 interacts with multiple cytoskeletal proteins in humans and mice (Fig. 2a). Thus, we hypothesized that accessory cytoskeletal elements might guide FABP5 surface localization. We conducted unbiased immunoprecipitation assays followed by mass spectrometry to identify FABP5-binding partners in mouse CD8^+^ T cells activated via CD3/CD28 (Fig. 2b). Consistent with the STRING predictions, we identified multiple cytoskeletal proteins that associate with FABP5, with Transgelin 2 (TAGLN2) demonstrating the highest representation (Fig. 2c). Interestingly, previous reports indicate that FABP5 and TAGLN2 coexist within macromolecular complexes conserved across multiple metazoans^29^. Indeed, we confirmed the endogenous FABP5-TAGLN2 interaction in mouse activated CD8^+^ T cells through co-immunoprecipitation assays (Fig. 2d). TAGLN2 is an actin-binding protein that stabilizes actin structures and participates in multiple actin-associated signaling pathways^30,31^. This small (22-kDa) protein has been shown to promote lipid uptake and utilization in adipocytes^32^, and has been implicated in T cell activation, adhesion, migration, and effector function^33^. Therefore, we hypothesized that TAGLN2 mediated FABP5 surface localization and fatty acid uptake in CD8^+^ T cells. We generated new transgenic mice where exon 3 of the *Tagln2* gene was flanked by two *loxp* sites (*Tagln2*^fl/fl^), and these mice were crossed with the *Cd4*^Cre^ strain to attain selective abrogation of TAGLN2 in T cells (Fig. 2e–g and Extended Data Fig. 2a). *Tagln2*^fl/fl^*Cd4*^Cre^ mice did not exhibit gross abnormalities and showed normal proportion of thymocytes and peripheral CD4^+^ and CD8^+^ T cells, including their corresponding subsets (Extended Data Fig. 2b–e). However, these conditional knockout mice exhibited a significant decrease in the proportion of splenic CD62L^high^CD44^high^ central memory CD8^+^ T cells (Extended Data Fig. 2b–e). In addition, TAGLN2-deficient CD8^+^ T cells stimulated via CD3/CD28 showed a drastic decrease in the expression of activation (CD44) and proliferation (Ki-67) markers (Fig. 2h,i). Strikingly, while the levels of total FABP5 remained unaltered (Fig. 2j), surface localization of FABP5 (Fig. 2k) and extracellular fatty acid uptake (Fig. 2l) were drastically reduced in TAGLN2-deficient activated CD8^+^ T cells, compared with their WT counterparts. These data indicate that the surface localization and function of FABP5 as a major importer of extracellular fatty acids in CD8^+^ T cells depends on TAGLN2.

To further confirm these findings, wild type (WT) or FABP5-deficient CD8^+^ T cells activated via CD3/CD28 were transfected with mRNAs encoding control ovalbumin (*Ctrl*-mRNA) or mouse TAGLN2 (*Tagln2*-mRNA), and their lipid uptake capacity was assessed thereafter (Fig. 2m). As expected, CD8^+^ T cells devoid of FABP5 showed lower lipid uptake than their WT counterparts (Fig. 2n). Notably, *Tagln2* overexpression bolstered extracellular fatty acid import in WT but not FABP5-deficient CD8^+^ T cells (Fig. 2n), and similar effects were observed when *Fabp5* was knocked-down in CD8^+^ T cells using siRNA (Extended Data Fig. 2f,g). Collectively, these data establish that FABP5 and TAGLN2 functionally cooperate to mediate optimal lipid import in CD8^+^T cells.

### Dysfunctional CD8^+^ T cells infiltrating OvCa lack TAGLN2.

**Extended Data Figure 3.**
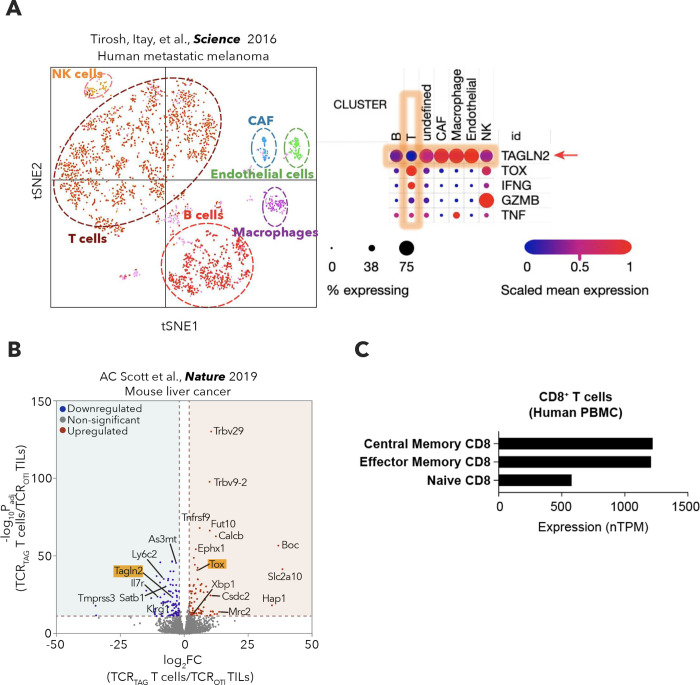
TAGLN2 downregulation in human and mouse exhausted CD8^+^ tumor-infiltrating T cells. **a**, *TAGLN2, TOX, IFNG, GZMB* and *TNF* mRNA expression were reanalyzed from single-cell RNA sequencing data generated from tumor-infiltrating lymphocytes isolated from melanoma patients (GSE72056) using the Broad Institute Single Cell Portal (https://singlecell.broadinstitute.org/single_cell). **b**, Volcano plot of differentially expressed genes in dysfunctional tumor-specific CD8^+^ T cells (TCR_TAG_) compared to non-tumor-specific CD8^+^ T cells (TCR_OT1_) from a mouse autochthonous liver tumor model (GSE126974). Selected differentially expressed genes with an adjusted *P* values <0.05 and Log_2_ Fold Change > 1 or −1 are highlighted. **c**, *TAGLN2* transcripts were shown in the indicated CD8^+^ T cell populations from human peripheral blood mononuclear cells (PBMC). The resulting transcript expression values calculated as normalized transcript per million (nTPM), resulting from the internal normalized pipeline (The Human Protein Atlas; proteinatlas.org).

**Figure 3.**
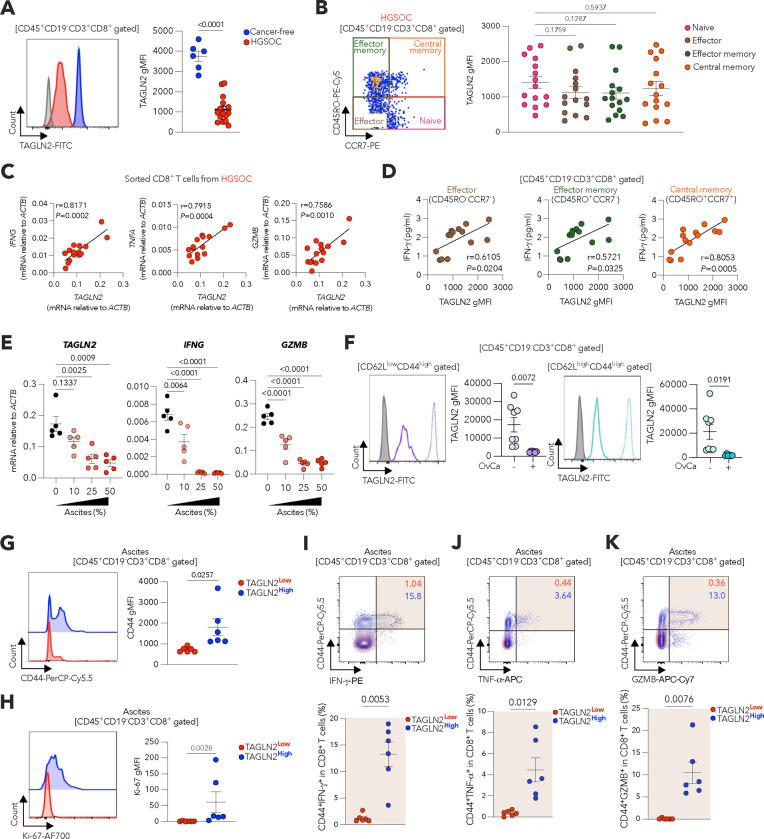
Status of TAGLN2 in OvCa-infiltrating CD8^+^ T cells. **a**, Representative FACS histograms and quantitative analysis of TAGLN2 protein levels in CD45^+^CD19^−^CD3^+^CD8^+^ T cells isolated from peripheral blood of cancer-free women (*n* = 6) or malignant ascites of HGSOC patients (*n* = 15). **b**, Representative FACS plot of CD8^+^ T cell-subset analysis from malignant ascites of HGSOC patients (left). TAGLN2 protein expression was quantified in naïve (pink), effector (brown), effector memory (green) and central memory (orange) CD8^+^ T cells, respectively (right) (*n* = 15). Cells were pre-gated on CD45^+^CD19^−^CD3^+^CD8^+^ T cells. naïve, CCR7^+^CD45RO^−^; effector, CCR7^−^CD45RO^−^; effector memory, CCR7^−^CD45RO^+^; central memory, CCR7^+^CD45RO^+^. **c**, Correlation analysis for *IFNG*, *TNFA* or *GZMB* versus *TAGLN2* mRNA in CD8^+^ T cells from malignant ascites of HGSOC patients of. Data were normalized to *ACTB* in all cases (*n* = 15). **d**, Correlation of IFN-g concentration versus levels of TAGLN2 in the indicated CD8^+^ T cell subsets in ascites of HGSOC patients (*n* =14). **e**, Naïve CD8^+^ T cells from peripheral blood of cancer-free women were activated via CD3/CD28 stimulation for 32 h and then incubated for 16 h with increasing amounts of HGSOC ascites supernatants (*n* = 5). Expression of *TAGLN2*, *IFNG,* and *GZMB* was assessed by qRT-PCR. Data were normalized to *ACTB.* **f**, Representative FACS histograms and quantitative analysis of TAGLN2 protein levels in effector (CD62L^low^CD44^high^) or central memory (CD62L^high^CD44^high^) CD8^+^ T cells from peritoneal wash of cancer-free mice (*n* = 8) or malignant ascites of female mice bearing ID8-*Defb29/Vegfa* OvCa (*n* = 6). **g,h**, Representative FACS histograms and quantitative analysis of CD44 (**g**) and Ki-67 (**h**) expression in TAGLN2^low^ or TAGLN2^high^ CD45^+^CD19^−^CD3^+^CD8^+^ T cells from malignant ascites of female mice bearing ID8-*Defb29/Vegfa* OvCa (*n* = 6 per group). **i-k**, Representative FACS plots and quantitative analysis of CD44^+^IFN-g^+^ (**i**), CD44^+^TNF-a^+^ (**j**) and CD44^+^GZMB^+^ (**k**) frequencies in TAGLN2^low^ or TAGLN2^high^ CD45^+^CD19^−^CD3^+^CD8^+^ T cells from the same mice described in (**g**) and (**h**). Data are presented as mean ± s.e.m. **a,f-h,** Two-tailed unpaired Student’s t-test. **b,e**, One-way ANOVA with Tukey’s multiple comparisons test. **c,d**, Spearman’s rank correlation test, Spearman coefficient (*r*) with exact *P-value* (two-tailed). **i-k**, Two-tailed paired Student’s t-test. Exact *P-values* are shown. The ‘*n*’ values represent biologically independent samples. gMFI, Geometric mean fluorescence intensity.

TAGLN2 has been implicated in T cell cytokine production and effector capacity in vitro^33^. Moreover, *Tagln2* is upregulated in functionally competent effector and memory CD8^+^ T cell subsets during viral infection^34,35^. Whether tumors silence TAGLN2 in infiltrating T cells to alter their lipid metabolism and protective activity is unknown. We analyzed previously published gene expression datasets^36,37^ and found marked *TAGLN2* downregulation in dysfunctional and exhausted CD8^+^ T cells infiltrating human metastatic melanoma and murine hepatocellular carcinoma (Extended Data Fig. 3a,b). Furthermore, we found that the intracellular levels of TAGLN2 were significantly lower in CD8^+^ T cells isolated from the ascites of HGSOC patients than in peripheral CD8^+^ T cells from cancer-free women (Fig. 3a). While activated T cells normally express higher TAGLN2 than their naïve counterparts (Extended Data Fig. 3c), effector and memory T cells present in the ascites of HGSOC patients demonstrated low TAGLN2 expression that was comparable to that of naïve T cells in the same microenvironment (Fig. 3b). The levels of *TAGLN2* positively correlated with the intrinsic expression of *IFNG*, *TNFA*, and *GZMB* transcripts in CD8^+^ T cells residing in the ascites of HGSOC patients (Fig. 3c). Furthermore, the amount of TAGLN2 in effector and memory CD8^+^ T cell subsets in this malignant fluid correlated with the local concentration of IFN-γ (Fig. 3d). Exposure to cell-free ascites supernatants from HGSOC patients drastically suppressed *TAGLN2*, *IFNG,* and *GZMB* expression in pre-activated CD8^+^ T cells obtained from peripheral blood of cancer-free women (Fig. 3e). In the ascites of mice with metastatic ID8-*Defb29/Vegfa* OvCa, effector (CD62L^low^CD44^high^) and central memory (CD62L^high^CD44^high^) CD8^+^ T cells demonstrated a marked reduction in TAGLN2 expression, compared with the same T cell subsets in peritoneal lavage from cancer-free mice (Fig. 3f). Notably, in this model, ascites-resident TAGLN2^high^ CD8^+^ T cells demonstrated higher effector capacity than their TAGLN2^low^ counterparts as evidenced by superior expression of CD44, Ki-67, IFN-γ, TNF-α, and Granzyme B (Fig. 3g–k). These results indicate that OvCa inhibits TAGLN2 expression in infiltrating CD8^+^ T cells and suggest that that loss of this cytoskeletal element is linked to intratumoral T cell malfunction.

### ER stress responses suppress TAGLN2 expression in CD8^+^ T cells.

**Extended Data Figure 4.**
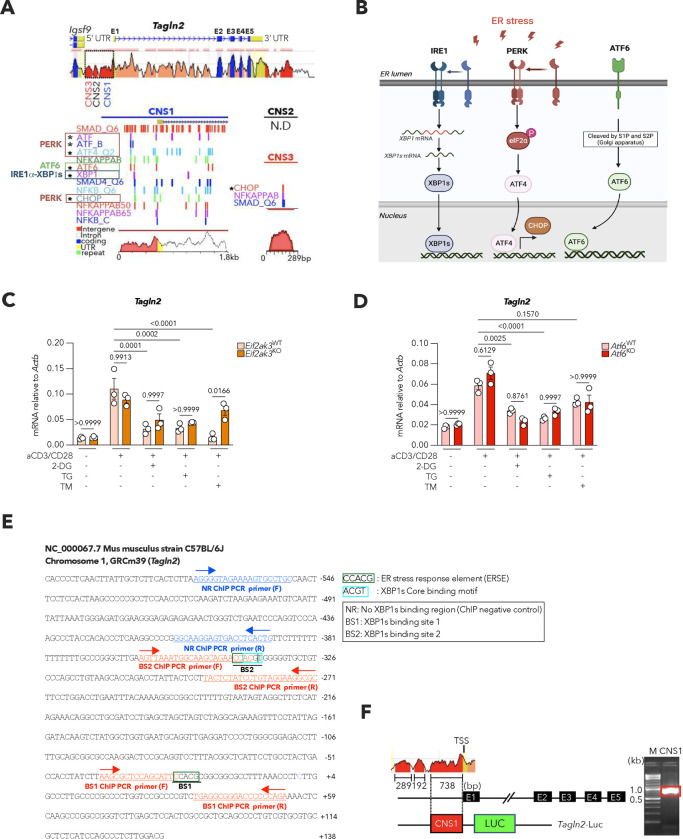
Putative transcription factor binding sites in the *Tagln2* promoter. **a**, ECR browser analysis of the mouse and human *Tagln2* locus is shown. The mouse genomic sequence was used as the base sequence on the x-axis. Schematic representation of the genomic positions of exons (E1–5) and putative binding sites of NF-kB and unfolded protein response (UPR) transcription factors in the *Tagln2* promoter regions, mainly in the CNS1 region. Asterisks denote UPR transcription factors. UTR, untranslated region. **b**, Schematic representation of ER stress sensors and their corresponding downstream transcription factors. **c,d**, Naïve CD8^+^ T cells isolated from *Eif2ak3*^fl/fl^ and *Eif2ak3*^fl/fl^*Vav1*^Cre^ mice (**c**) or *Atf6*^fl/fl^ and *Atf6*^fl/fl^*Vav1*^Cre^ mice (**d**) were cultured under the indicated conditions. Expression of the *Tagln2* transcript was determined by RT-qPCR, and data were normalized to endogenous levels of *Actb* in each sample (*n* = 3 per condition and genotype). **e**, Sequences of mouse *Tagln2* promoter from −646 to +138. XBP1s binding sites (Score > 10) are marked as BS1 and BS2. Location of ChIP-PCR primers (F/R) is indicated. **f**, *Tagln2* promoter construct used for luciferase reporter assays. **c,d**, One-way ANOVA. *P*<0.05 is considered to be statistically significant and exact *P-values* are shown. The ‘*n*’ values represent biologically independent samples.

**Figure 4.**
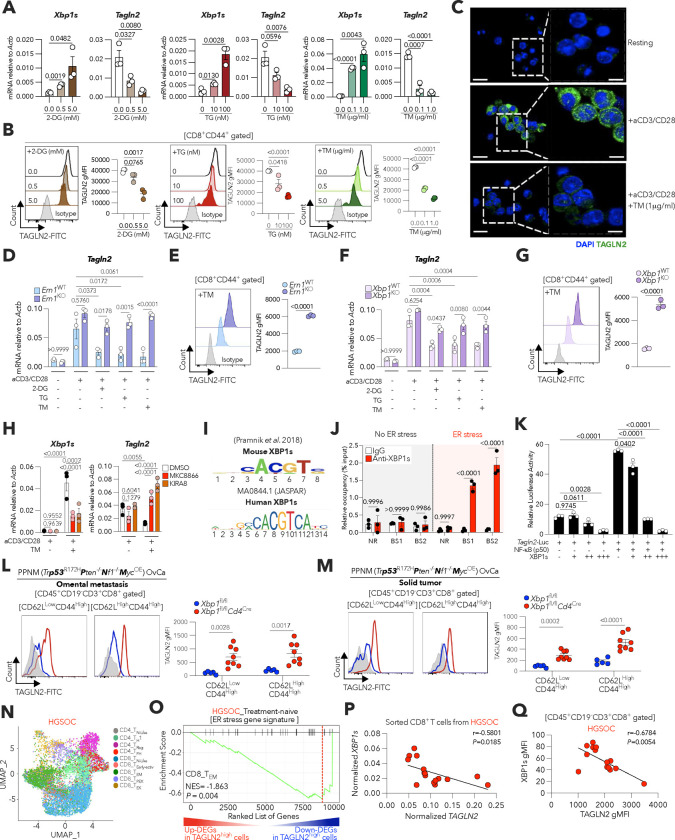
XBP1s restrains TAGLN2 expression in ER-stressed CD8^+^ T cells. **a**, Pre-activated CD8^+^ T cells from WT C57BL/6J mice were treated with 2-Deoxy-D-glucose (2-DG), Tunicamycin (TM) or Thapsigargin (TG) at the indicated concentrations. *Xbp1s* and *Tagln2* expression were determined via qRT-PCR 16 h after 2-DG or TM treatment, and 6 h post TG exposure. Data were normalized to *Actb* in each sample (*n* = 3 per condition). **b**, TAGLN2 protein expression levels determined by FACS (*n* = 3 per group) in the same samples described in (**a**). **c**, Representative confocal images for TAGLN2 expression in CD8^+^ T cells from WT C57BL/6J mice under the indicated conditions from two independent experiments. **d-g**, Naïve CD8^+^ T cells isolated from *Ern1*^fl/fl^ or *Ern1* ^fl/fl^ *Cd4*^Cre^ mice (**d,e**) or *Xbp1*^fl/fl^ or *Xbp1*^fl/fl^*Cd4* ^Cre^ mice (**f,g**) were cultured under the indicated conditions. Expression of the *Tagln2* transcript was determined by RT-qPCR, and data were normalized to endogenous levels of *Actb* in each sample (**d,f**) (*n* = 3 per condition and genotype). Representative FACS histograms and quantitative analysis of TAGLN2 protein levels in CD8^+^CD44^+^ T cells under the indicated conditions (**e,g**) (*n* = 3 per genotype). **h**, Pre-activated CD8^+^ T cells from WT C57BL/6J mice were stimulated via CD3/CD28 for 16 h in the absence or presence of TM (1 μg/ml). White bars, DMSO; Red bars, MKC8866 (2 μM); Orange bars, KIRA8 (1 μM). Expression of *Xbp1s* and *Tagln2* transcripts were determined by RT-qPCR, and data were normalized to endogenous levels of *Actb* in each sample (*n* = 3 per condition). **i**, Conserved XBP1s-binding motifs (CACGTC) from mouse (top) and human (bottom) are shown. **j**, Pre-activated CD8^+^ T cells from WT C57BL/6J mice were stimulated via CD3/CD28 for 16 h in the absence or presence of the ER stressor TM (1 μg/ml). ChIP assays were performed using anti-XBP1s or isotype control antibodies. qRT-PCR was used to determine XBP1s occupancy at two XBP1s-binding sites (BS1 and BS2) in *Tagln2* promoter regions under the conditions tested. No XBP1s binding region (NR) was used as a negative control. ChIP-quantitative PCR assays were performed using T cells from three independent mice (*n* = 3 per condition). **k**, *Tagln2* promoter-luciferase construct (−738 to +134) was co-transfected with the combination of NF-kB (p50) and XBP1s expressing vectors in HEK-293 T cells. Lysates were prepared 48 h after transfection, and luciferase activities were measured with the firefly luciferase activities normalized to renilla luciferase activities (*n* = 3). **l,m**, Representative FACS histograms and quantitative analysis of TAGLN2 protein levels in effector (CD62L^low^CD44^high^) or central memory (CD62L^high^CD44^high^) CD8^+^ intratumoral T cells in omentum (**l**) and solid tumor (**m**) from female mice of the indicated genotypes bearing PPNM-based HGSC for 40 days (*Xbp1*^fl/fl^, *n* = 5; *Xbp1*^fl/fl^*Cd4* ^Cre^, *n* = 8). **n**, UMAP plot of T cell subtypes from 11 HGSOC treatment-naïve human tumor specimens. **o**, GSEA enrichment plots showing downregulation of ER stress gene signature in *TAGLN2*^hi^ CD8^+^ tumor-infiltrating effector memory T cells (T_EM_). NES, normalized enrichment score. **p**, Correlation analysis for *Xbp1s* versus *TAGLN2* mRNA expression levels in CD8^+^ T cells residing in the ascites of HGSOC patients. Data were normalized to *ACTB* (*n* = 16). **q**, Correlation of XBP1s versus TAGLN2 protein expression in CD45^+^CD19^−^CD3^+^CD8^+^ T cells from the ascites of HGSOC patients (*n* =14). Data are presented as mean ± s.e.m. **a,b,d,f,h,k,** One-way ANOVA with Tukey’s multiple comparisons test. **e,g,j,l,m**, Two-tailed unpaired Student’s t-test. **p,q**, Spearman’s rank correlation test, Spearman coefficient (*r*) with exact *P-value* (two-tailed). *P*<0.05 is considered statistically significant and exact *P-values* are shown. The ‘*n*’ values represent biologically independent samples. gMFI, Geometric mean fluorescence intensity.

We sought to define the molecular mechanisms mediating TAGLN2 repression in OvCa-infiltrating CD8^+^ T cells. We identified three conserved noncoding sequences (CNS1, CNS2, and CNS3) within the 5’ promoter region of the *Tagln2* locus that were highly associated with transcription factor binding sites and other *cis*-acting regulatory elements (Extended Data Fig. 4a). Intriguingly, CNS1 (−738 to +134) contained multiple putative binding sites for transcription factors governing the endoplasmic reticulum (ER) stress response, including XBP1s, ATF4, and ATF6 (Extended Data Fig. 4a). These elements are induced upon activation of the ER-resident stress sensors inositol-requiring enzyme-1α (IRE1α), protein kinase RNA-like endoplasmic reticulum kinase (PERK), and activating transcription factor 6 (ATF6), respectively^38^ (Extended Data Fig. 4b). Since dysregulated ER stress responses promote T cell malfunction and immune escape in diverse tumor types, including OvCa^17,18,39^, we evaluated whether ER stress-inducing conditions altered *Tagln2* expression in activated CD8^+^ T cells. Exposure to classical ER stressors such as 2-Deoxy-D-glucose (2-DG), thapsigargin (TG), or tunicamycin (TM) induced the canonical ER stress response marker *Xbp1s* while markedly decreasing the expression of *Tagln2* (Fig. 4a). FACS analyses confirmed reduced intracellular TAGLN2 levels in activated CD8^+^ T cells experiencing ER stress (Fig. 4b), and these results were further validated by confocal microscopy (Fig. 4c).

To determine the arms of the ER stress response mediating *Tagln2* repression, similar experiments were conducted using transgenic CD8^+^ T cells independently lacking IRE1α, PERK, or ATF6. Signaling through PERK or ATF6 did not play a major role in regulating *Tagln2* expression in ER-stressed CD8^+^ T cells (Extended Data Fig. 4c,d). In contrast, IRE1α-deficient CD8^+^ T cells facing ER stress failed to downregulate *Tagln2* (Fig. 4d) and maintained higher TAGLN2 protein expression (Fig. 4e) than their WT counterparts under the same condition. Upon activation, IRE1α excises a 26-nucleotide fragment from the *Xbp1* mRNA, generating a spliced isoform that codes for the functionally active transcription factor XBP1s^40^ (Extended Data Fig. 4b). Notably, XBP1s-deficient CD8^+^ T cells also preserved *Tagln2* transcription and protein expression under ER stress (Fig. 4f,g). Treatment with the selective IRE1α pharmacological inhibitors MKC8866^41^ or KIRA8^42^, which prevent XBP1s generation, also enhanced *Tagln2* expression in activated CD8^+^ T cells undergoing ER stress (Fig. 4h). Hence, the IRE1α-XBP1s arm of the ER stress response operates as a dominant negative regulator of TAGLN2 expression in activated CD8^+^ T cells.

XBP1s is a multitasking transcription factor that controls gene expression in a cell-specific and context-dependent manner^39,43^. We examined whether XBP1s could bind the *Tagln2* promoter to directly alter its transcription. Confirming our initial analysis (Extended Data Fig. 4a), putative XBP1s core binding motif and ER stress response element (ERSE) sequences were found in the *Tagln2* promoter (Fig. 4i and Extended Data Fig. 4e). Chromatin immunoprecipitation (ChIP) followed by PCR (ChIP-PCR) experiments demonstrated robust XBP1s binding to the promoter regions identified only in pre-activated CD8^+^ T cells experiencing ER stress (Fig. 4j). Interestingly, CNS1 also contained multiple binding motifs for NF-kB (Extended Data Fig. 4a), which had been shown to act as a positive regulator of *Tagln2* expression in macrophages^44^. Thus, we used luciferase reporter assays (Extended Data Fig. 4f) to test whether XBP1s inhibited NF-kB-directed transcription of *Tagln2*. Potent luciferase reporter activity driven by the *Tagln2* promoter was observed upon NF-kB expression (Fig. 4k). Yet, this transactivation was significantly and dose-dependently impaired upon introduction of XBP1s-encoding plasmids (Fig. 4k). These data uncover ER stress-induced XBP1s as a transcriptional repressor of *Tagln2*.

**Extended Data Figure 5.**
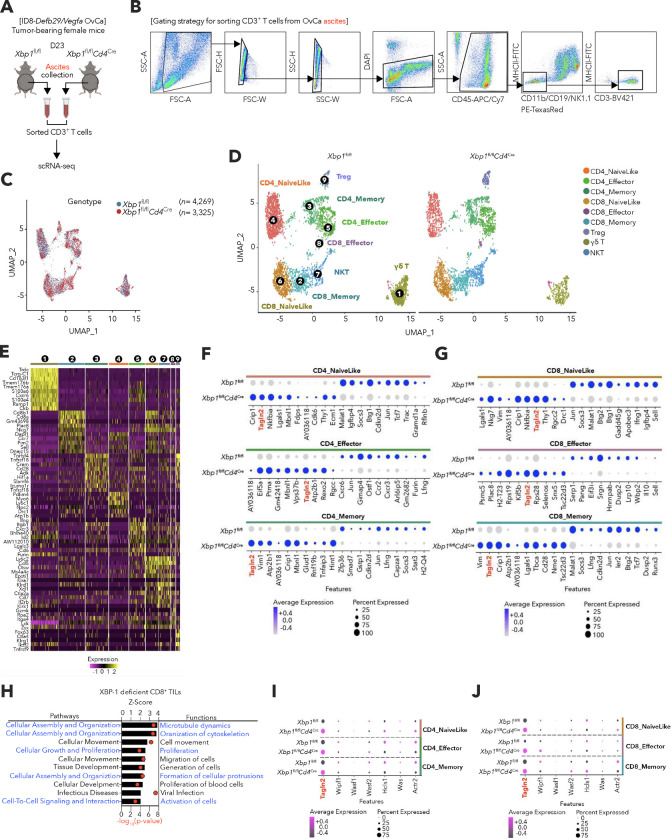
Elevated *Tagln2* expression in multiple intratumoral CD4^+^ and CD8^+^ T cell subsets lacking XBP1s. **a**, Schematic illustrating sample processing and experimental workflow. **b**, FACS sorting strategy for scRNA-sequencing. **c**, UMAP colored by genotype classifications of *Xbp1*^fl/fl^ (blue, *n* = 4,269 cells) and *Xbp1*^fl/fl^*Cd4*^Cre^ (red, *n* = 3,325 cells). **d**, UMAP plot visualization of different T cell clusters colored by cell type. **e**, Heatmap showing the top 10 marker genes of the subclusters. **f,g**, Dot plots show top 10 upregulated or downregulated genes in CD4^+^ (**f**) or CD8^+^ (**g**) intratumoral T cell clusters from XBP1s-deficient compared to WT control. The colors represent the average expression levels, and dot sizes represent the percentage expression of each gene in the indicated clusters. **h**, Enriched cellular pathways and functions in XBP1s-deficient CD8^+^ T cells at tumor sites. Z-scores greater than 2 indicate pathways and functions predicted to be significantly increased in XBP1s-deficient CD8^+^ T cells. **i,j**, Dot plot analysis showing the expression levels and distribution of seven major cytoskeletal genes (*Tagln2, Wipf1, Wasf1, Wasf2, Hcls1, Was* and *Actr2*) identified T cells across the indicated cellular clusters identified in (**d**).

We next evaluated whether IRE1α-XBP1s signaling regulates *Tagln2* expression in OvCa-infiltrating CD8^+^ T cells experiencing pathological ER stress in the tumor microenvironment^17^. To this end, we performed single-cell RNA sequencing (scRNA-seq) analyses of total CD45^+^CD3^+^ T cells sorted from the ascites of *Xbp1*^fl/fl^ or *Xbp1* ^fl/fl^*Cd4*^Cre^ mice bearing advanced ID8-*Defb29*/*Vegfa* OvCa (Extended Data Fig. 5a–e). *Tagln2* was significantly upregulated in multiple ascites-infiltrating CD4^+^ and CD8^+^ T cell subsets lacking XBP1s, compared with their WT counterparts (Extended Data Fig. 5f,g). Of note, downstream cellular functions such as microtubule dynamics, organization of cytoskeleton, and proliferation and activation of cells, were predicted to be enhanced in ascites-infiltrating CD8^+^ T cells lacking XBP1s that maintain superior *Tagln2* expression (Extended Data Fig. 5h). When compared to other key cytoskeletal elements, only *Tagln2* exhibited a distinct increase in various ascites-infiltrating CD4^+^ and CD8^+^ T cell subsets devoid of XBP1s (Extended Data Fig. 5i,j).

**Extended Data Figure 6.**
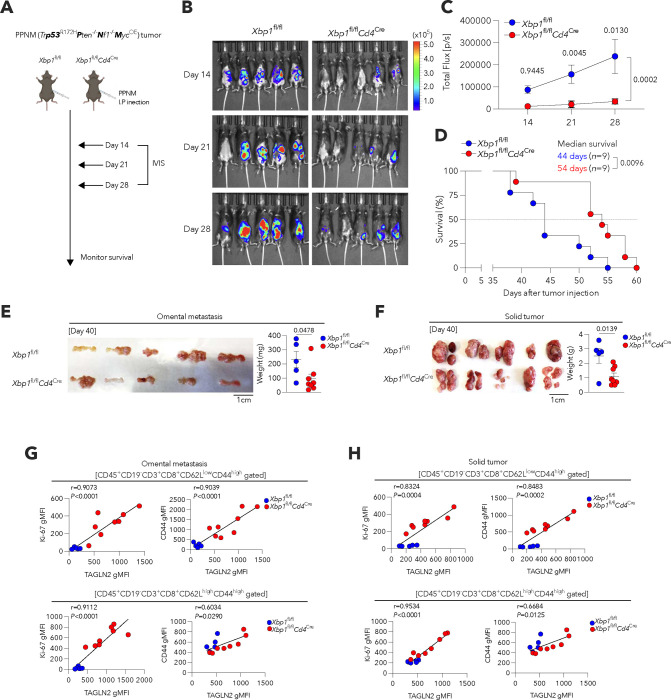
Selective loss of XBP1s in T cells delays malignant tumor progression and enhances TAGLN2, Ki-67, and CD44 expression in PPNM tumor-infiltrating CD8^+^ T cells. **a**, Experimental scheme for mice of the indicated genotypes implanted with luciferase-expressing PPNM cancer cells. **b,c**, Assessment of peritoneal tumor burden over time in mice of the indicated genotypes (**b**) and quantification of bioluminescent signal for the same mice of the indicated genotype at different time points (*n* = 5 per genotype) (**c**). **d**, Overall survival curves for PPNM-bearing female mice of the indicated genotypes (*n* = 9 per genotype). **e,f**, Representative images of omentum (**e**) and solid tumors (**f**) from female mice of the indicated genotypes bearing PPNM-based HGSC for 40 days. Weight of omentum (**e**) and solid tumors (**f**) was determined in each group (*Xbp1*^fl/fl^, *n* = 5; *Xbp1*^fl/fl^*Cd4* ^Cre^, *n* = 8). **g,h**, Correlation of protein expression levels of TAGLN2 versus either Ki-67 or CD44 in the indicated intratumoral CD8^+^ T cell subsets in omentum (**g**) and solid tumor (**h**) from female mice of indicated genotypes bearing PPNM-bearing HGSC for 40 days (*Xbp1*^fl/fl^, *n* = 5; *Xbp1*^fl/fl^*Cd4* ^Cre^, *n* = 8). Data are presented as mean ± s.e.m. **c**, One-way ANOVA with Tukey multiple comparisons test. **d**, Log-rank test for survival. **e,f**, Two-tailed unpaired Student’s t-test. **g,h**, Spearman’s rank correlation test, Spearman coefficient (*r*) with exact *P-value* (two-tailed). *P*<0.05 is considered to be statistically significant and exact *P-values* are shown. The ‘*n*’ values represent biologically independent samples. gMFI, Geometric mean fluorescence intensity.

To confirm these results, we used the PPNM model of high-grade serous tubo-ovarian cancer (HGSC) that encompasses the most common genetic abnormalities observed in human HGSOC^45^. Consistent with our prior findings^17^, T cell-intrinsic XBP1s also facilitated metastatic OvCa progression in this independent tumor system (Extended Data Fig. 6a–f). Furthermore, XBP1s-deficient effector (CD62L^low^CD44^high^) and central memory (CD62L^high^CD44^high^) CD8^+^ T cells infiltrating PPNM-derived omental and peritoneal tumors demonstrated higher expression of TAGLN2, Ki-67, and CD44 than their XBP1s-sufficient (*Xbp1*^fl/fl^) counterparts (Fig. 4l,m and Extended Data Fig. 6g,h).

We conducted gene-set enrichment analyses (GSEA) of scRNA-seq data generated from patient-derived HGSOC specimens^46^ to further validate the findings. *TAGLN2*^high^ effector memory CD8^+^ T cells infiltrating these human tumors demonstrated reduced expression of multiple ER stress response gene markers, including *XBP1* and its canonical target genes (Fig. 4n,o). Notably, TAGLN2 mRNA and protein expression inversely correlated with the intrinsic levels of XBP1s in CD8^+^ T cells residing in the ascites of HGSOC patients (Fig. 4p,q). Taken together, these data indicate that ER stress-driven IRE1α-XBP1s blunts TAGLN2 expression in intratumoral CD8^+^ T cells to enforce their dysfunctional state.

### TAGLN2 overexpression enhances lipid uptake and mitochondrial respiration in ER-stressed CD8^+^ T cells.

**Figure 5.**
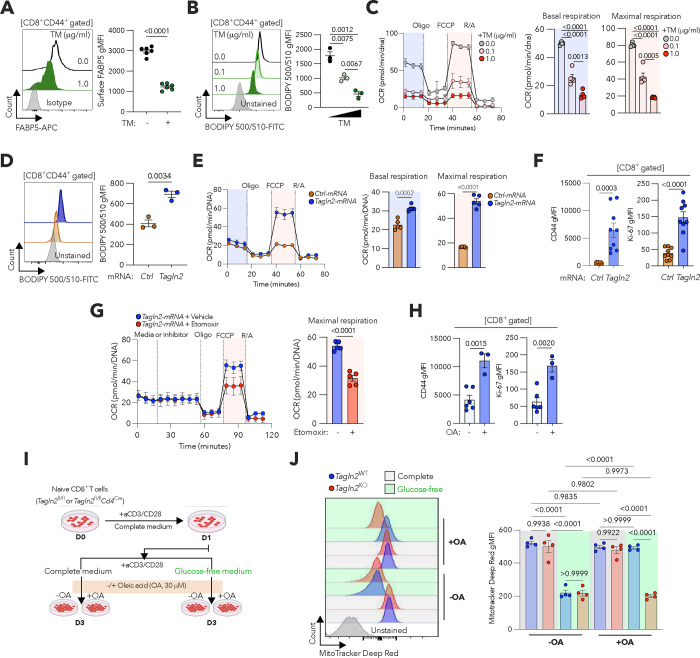
TAGLN2 overexpression rescues the bioenergetic defects of ER-stressed CD8^+^ T cells. **a-c**, Pre-activated CD8^+^ T cells from WT C57BL/6J mice were stimulated via CD3/CD28 for 16 h in the presence of TM at the indicated concentrations. Representative FACS histograms and quantitative analysis of cell surface FABP5 expression (**a**) and lipid uptake (**b**) in CD8^+^CD44^+^ T cells. **c**, Representative OCR plots (left) and quantification (right) of basal (blue) and maximal respiration (red) are shown. Data were normalized to total genomic DNA content in each condition (*n* = 5). **d-f**, Naïve CD8^+^ T cells from WT C57BL/6J mice were stimulated via CD3/CD28 for 24 h and then Neon-electroporated with either Control (*Ctrl*) or mouse *Tagln2* mRNAs. Electroporated T cells were maintained under CD3/CD28 stimulation for an additional 32 h and then treated with the ER stressor TM (1 μg/ml) for 16 h. (**d**) Representative FACS histograms and quantitative analysis of lipid uptake. (**e**) Representative OCR plots (left) are shown. Rates of basal respiration (middle) and maximal respiratory capacity (right) were quantified and normalized to total genomic DNA content (*n* = 5 per condition). (**f**) Expression levels of CD44 (left) and Ki-67 (right) in CD8^+^ T cells electroporated with the indicated mRNAs and exposed to TM (*n* = 9 per condition). **g**, OCR of TM-treated *Tagln2*-overexpressing CD8^+^ T cells in response to media (vehicle) or etomoxir injection. The maximal respiratory capacity was quantified and normalized to total genomic DNA content (*n* = 5 per condition). **h**, Expression levels of CD44 (left) and Ki-67 (right) in TM-treated *Tagln2*-overexpressing CD8^+^ T cells in the absence or presence of exogenous oleic acid (OA, 30 μM). **i,j**, WT or TAGLN2-deficient naïve CD8^+^ T cells from spleen and lymph nodes were activated via CD3/CD28 stimulation in complete medium for 24 h, followed by 48 h culture with or without oleic acid in either complete- or glucose-free medium. (**i**) Experimental scheme. (**j**) Representative FACS plots and quantitative analysis of mitochondrial membrane potential analyzed by MitoTracker Deep Red staining (*n* = 4 per condition). Data are presented as mean ± s.e.m. **a,d-h,** Two-tailed unpaired Student’s t-test. **b,c,j**, One-way ANOVA with Tukey’s multiple comparisons test. *P*<0.05 is considered statistically significant and exact *P-values* are shown. The ‘*n*’ values represent biologically independent samples. gMFI, Geometric mean fluorescence intensity.

Extracellular fatty acids imported by transporters such as FABP5 are catabolized via fatty acid β-oxidation (FAO) to fuel mitochondrial respiration and generate ATP^47^. We hypothesized that ER stress-driven repression of TAGLN2 disrupted the FABP5-FAO bioenergetic axis. Indeed, pre-activated CD8^+^ T cells treated with the ER stressor tunicamycin showed diminished FABP5 surface expression (Fig. 5a) and impaired lipid uptake capacity (Fig. 5b) compared with their non-stressed counterparts. Moreover, CD8^+^ T cells undergoing ER stress demonstrated a dose-dependent decrease in their basal and maximal oxygen consumption rates (Fig. 5c). We tested whether increasing *Tagln2* levels via mRNA electroporation could restore the metabolic defects caused by ER stress. Strikingly, *Tagln2*-rescued CD8^+^ T cells facing ER stress demonstrated superior lipid uptake (Fig. 5d), enhanced mitochondrial respiration (Fig. 5e), and increased expression of activation (CD44) and proliferation (Ki-67) markers (Fig. 5f), compared with their counterparts transfected with a control mRNA encoding ovalbumin. Treatment with the CPT1α inhibitor etomoxir rendered *Tagln2*-overexpressing CD8^+^ T cells unable to enhance mitochondrial respiration under ER stress (Fig. 5g), confirming that FAO mediates these effects. Exposure to oleic acid, one of the most abundant free-fatty acids in the ascites of HGSOC patients^48^, bolstered CD44 and Ki-67 expression in ER-stressed CD8^+^ T cells overexpressing *Tagln2* (Fig. 5h), denoting improved usage of these extracellular lipids to support their effector programs. Thus, we next tested whether TAGLN2 enables the use of these fatty acids to sustain mitochondrial activity in activated CD8^+^ T cells experiencing glucose restriction (Fig. 5i), which is a prevalent condition in the tumor milieu. In the absence of exogenous oleic acid, glucose deprivation drastically reduced the mitochondrial membrane potential of CD8^+^ T cells irrespective of TAGLN2 status (Fig. 5j). Notably, oleic acid supplementation fully restored the mitochondrial membrane potential of WT but not TAGLN2-deficient CD8 T cells under glucose restriction (Fig. 5j). Hence, ER stress blunts CD8^+^ T cell mitochondrial respiration by suppressing TAGLN2-mediated FAO. In addition, TAGLN2 promotes CD8^+^ T cell metabolic fitness by enabling the uptake and usage of extracellular fatty acids that sustain mitochondrial activity in the absence of primary carbon sources such as glucose.

### Preserving TAGLN2 improves cellular immunotherapy in metastatic OvCa.

**Extended Data Figure 7.**
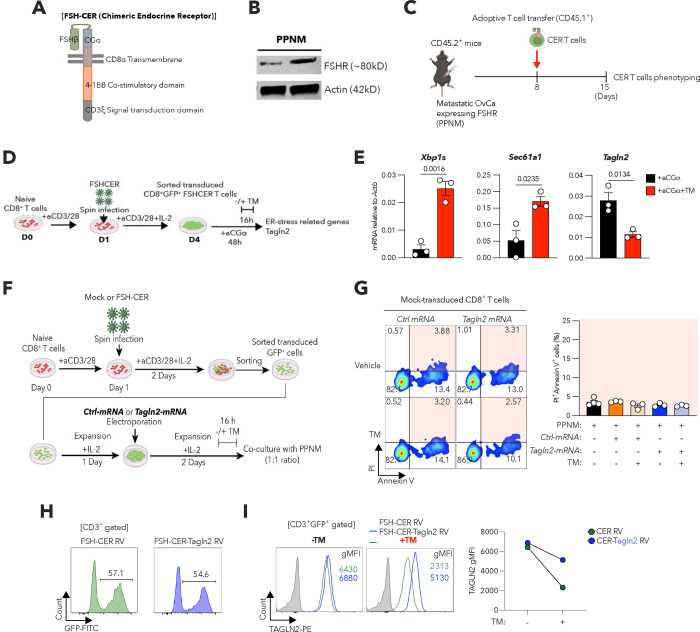
ER stress responses in CER T cells. **a,** Main features of the FSH-CER retroviral construct. **b**, Expression of the follicle-stimulating hormone receptor (FSHR) by PPNM cancer cells was determined by immunoblot analysis in which β-actin was used as loading control. **c**, Experimental scheme for analysis of CER T cells at tumor locations 7 days after adoptive transfer. **d,e**, CD8^+^GFP^+^ sorted CER T cells were stimulated with recombinant chorionic gonadotropin alpha (CGα) in the absence or presence of TM (**d**). *Xbp1s, Sec61a1*, and *Tagln2* expression was determined via qRT-PCR. Data were normalized to *Actb* (*n* = 3 per condition) (**e**). **f,** Experimental scheme to assess the effect of ER stress in Mock or FSH-CER transfuced CD8^+^ T cells. **g**, CD8^+^GFP^+^ sorted Mock transduced T cells were electroporated with the indicated mRNAs and then treated with vehicle or TM for 16 h. T cells were washed to remove TM and then cocultured with PPNM cancer cells at a 1:1 ratio. Cancer cell death was assessed by Annexin V and PI staining by FACS 18 h later. Representative FACS plots and quantitative analysis (*n* = 3–4 per condition). **h,** FACS-based analysis to assess transduction efficiency using GFP expression as a marker. **i**, CER or CER-Tagln2 T cells were incubated in the presence or absence of TM for 16 h and TAGLN2 protein expression was measured by FACS. Data are presented as mean ± s.e.m. **e**, Two-tailed unpaired Student’s t-test. *P*<0.05 is considered to be statistically significant and exact *P-values* are shown. The ‘*n*’ values represent biologically independent samples. gMFI, Geometric mean fluorescence intensity.

Adoptive T cell immunotherapies, including chimeric antigen receptor (CAR) T cells, have shown limited success against solid malignancies, especially OvCa^14,16^. We hypothesized that tumor-induced suppression of TAGLN2 limits the therapeutic efficacy of CAR T cells in hosts with metastatic OvCa. To test this, we exploited T cells expressing chimeric endocrine receptors (CERs) that use the two subunits of the follicle-stimulating hormone (FSH) to target and kill FSH-receptor positive (FSHR^+^) ovarian tumors^49^. In this system, T cells are transduced with a vector that encodes a chimeric receptor of the full-length of the b subunit of the FSH hormone, linked by a glycine/serine spacer, in frame with a CD8a transmembrane domain, the intracellular domain of co-stimulatory 4-1BB, and CD3ζ (Extended Data Fig. 7a)^49^. Importantly, these CER T cells are undergoing clinical testing in patients with advanced OvCa (NCT05316129).

**Figure 6.**
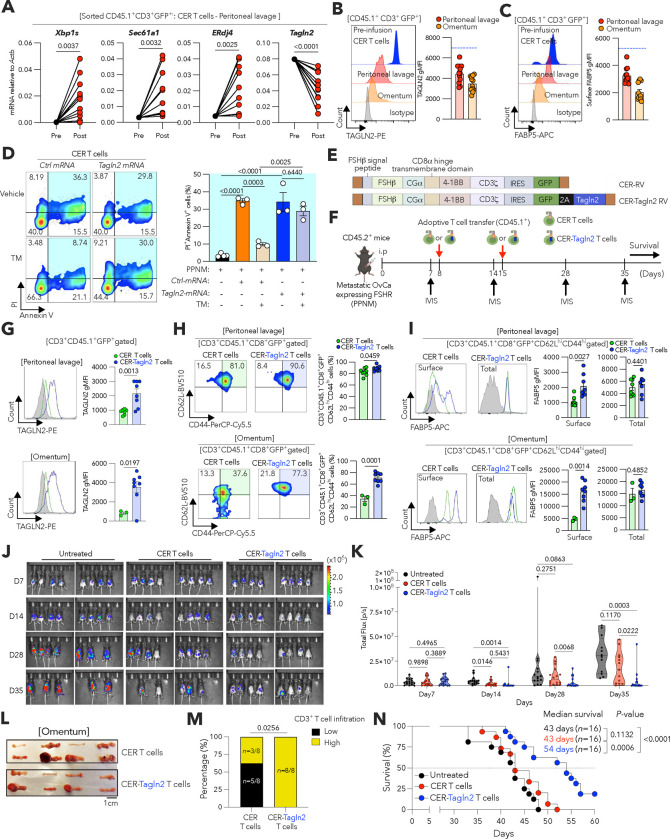
Preserving TAGLN2 enhances the therapeutic effects of CER T cells in OvCa. **a-c**, CD45.1^+^CER T cells were isolated from the indicated tumor sites seven days after adoptive transfer into WT C57BL/6J female mice developing PPNM HGSC. (**a**) *Xbp1s, Sec61a1, ERdj4,* and *Tagln2* expression in pre- or post-infusion CD45.1^+^CD3^+^GFP^+^ CER T cells was determined via qRT-PCR. Data were normalized to *Actb* (*n* = 12). Representative FACS histograms and quantitative analysis of TAGLN2 (**b**) and surface FABP5 (**c**) protein levels in gated CD45.1^+^CD3^+^GFP^+^ T cells from peritoneal lavage or omentum (*n* = 12 per group). Blue dotted lines in the bar graph represent the expression of TAGLN2 and surface FABP5 in pre-infusion CER T cells, respectively. **d**, CER T cells were electroporated with the indicated mRNAs and then treated with vehicle or TM (1 μg/ml) for 16 h. T cells were washed to remove TM and then cocultured with PPNM cancer cells at a 1:1 ratio. Cancer cell death was assessed via Annexin V and PI staining by FACS 18 h later. Representative FACS plots and quantitative analysis (*n* = 3–4 per condition). **e**, Schematics of retroviral CER expression constructs. FSHβ, Follicle-stimulating hormone beta subunit. CGα, chorionic gonadotropin alpha subunit. IRES, Internal ribosome entry site. GFP, Green fluorescent protein. **f**, Experimental scheme for adoptive transfer of CER or CER-Tagln2 T cells into PPNM tumor-bearing WT C57BL/6J female mice. **g-i**, Expression of TAGLN2 protein (**g**), frequencies of CD62L^hi^CD44^hi^ (central memory) (**h**), and cell surface and total levels of FABP5 protein (**i**) were assessed in the indicated CER T cell populations from peritoneal lavage or omentum at day 21 of tumor development (7 days after the second T cell infusion). Representative FACS histograms and quantitative analysis are shown (*n* = 3–8 mice per group). **j,k**, Peritoneal carcinomatosis in female mice bearing PPNM-based HGSC and treated with the indicated CER T cells. Representative bioluminescence images of PPNM tumors over time (**j**) and quantification of peritoneal tumor burden (**k**) in the indicated groups (*n* = 16 mice per group). **l,m**, Representative images of omentum (**l**), and quantification of CD3^+^ T cell infiltration into omental samples (**m**) in the indicated female mice bearing PPNM tumors (*n* = 8 per group). **n**, Overall survival rates for the mice described in (**j,k**) (*n* = 16 per group). Data are presented as mean ± s.e.m. **a,** Two-tailed paired Student’s t-test. **g-I,** Two-tailed unpaired Student’s t-test. **d,k,** One-way ANOVA with Tukey’s multiple comparisons test. **m**, Fisher’s exact test was used to determine the association between the two categorical variables. **n**, Log-rank test for survival. *P*<0.05 is considered statistically significant and exact *P-values* are shown. The ‘*n*’ values represent biologically independent samples. gMFI, Geometric mean fluorescence intensity.

Female mice developing PPNM-based ovarian tumors that inherently express the FSHR (Extended Data Fig. 7b) were intraperitoneally infused with CER T cells, and transferred T cells were sorted from tumor locations 7 days later based on the differential expression of congenic markers (Extended Data Fig. 7c). Notably, CER T cells recovered from the peritoneal cavity of tumor-bearing mice demonstrated upregulation of *Xbp1s* and the canonical XBP1s-target genes *Sec61a1*a and *ERdj4*, which was accompanied by marked *Tagln2* repression, compared with their counterparts prior to infusion (Fig. 6a). Consistently, CER T cells isolated from this anatomical location and metastatic omental lesions also demonstrated a profound decrease in TAGLN2 expression (Fig. 6b), as well as reduced levels of surface FABP5 (Fig. 6c), compared with the infusion product. These data demonstrate that CER T cells transferred into mice with metastatic OvCa undergo ER stress responses in situ that are accompanied by reduced TAGLN2 expression and impaired FABP5 surface localization. We therefore tested whether ER stress-driven repression of TAGLN2 compromised the tumoricidal activity of CER T cells. Tunicamycin-induced ER stress inhibited *Tagln2* expression in CER T cells (Extended Data Fig. 7d,e) and drastically reduced their cytotoxic capacity towards PPNM OvCa cells (Fig. 6d and Extended Data Fig. 7f,g). Strikingly, the killing capacity of ER-stressed CER T cells was restored upon electroporation with *Tagln2* but not control mRNAs (Fig. 6d). Hence, we surmised that preserving TAGLN2 expression in CER T cells could be used to enhance their therapeutic effects in the hostile OvCa microenvironment.

We subcloned the *Tagln2* cDNA downstream of the CER construct (CER-Tagln2 RV, Fig. 6e). This enabled simultaneous and constitutive expression of the CER and TAGLN2 upon transduction, and the maintenance of high TAGLN2 levels in T cells facing ER stress (Extended Data Fig. 7h,i). Thus, we next evaluated the status and therapeutic efficacy of TAGLN2-overexpressing CER T cells in mice bearing PPNM-based HGSC (Fig. 6f). Immunophenotyping analyses conducted 7 days after the second T cell infusion (day 21 of tumor development) demonstrated that CER-Tagln2 T cells infiltrating the omentum and peritoneal cavity maintained high TAGLN2 expression, while unmodified CER T cells present at the same tumor sites exhibited minimal production of this cytoskeletal element (Fig. 6g). Of note, preserving TAGLN2 expression increased the proportion of central memory (CD8^+^CD44^+^CD62L^+^) CER T cells in the peritoneal cavity and omentum (Fig. 6h) while augmenting their surface levels of FABP5 (Fig. 6i). Accordingly, TAGLN2-overexpressing CER T cells demonstrated improved capacity to control metastatic HGSC progression, as evidenced by reduced peritoneal carcinomatosis (Fig 6j,k) and enhanced T cell infiltration into metastatic omental lesions (Fig. 6l,m), compared with their control counterparts devoid of TAGLN2. Adoptive immunotherapy using unmodified CER T cells failed to confer a survival benefit in mice developing these aggressive tumors (Fig. 6n). Strikingly, however, treatment with TAGLN2-overpressing CER T cells significantly prolonged the overall survival of mice with metastatic disease (Fig. 6n). These data demonstrate that advanced ovarian tumors limit the protective capacity of adoptively transferred CER T cells by abrogating TAGLN2, and that preserving the expression of this cytoskeletal factor enables CER T cells to bypass the detrimental effects of tumor-induced ER stress, conferring improved therapeutic effects against metastatic disease.

Tumors create hostile microenvironments that impede the development and maintenance of effective anti-cancer immunity. Yet, how intratumoral immune cells integrate and interpret persistent stress signals in this harsh milieu remains incompletely understood. Here, we present experimental evidence indicating that ER stress responses cripple anti-tumor T cell function by disabling TAGLN2-coordinated immunometabolic programs. We propose that TAGLN2 operates as a central gatekeeper of CD8^+^ T cell lipid metabolic programming by enabling FABP5-driven import of fatty acids that fuel T cell mitochondrial respiration and effector function. Preserving the TAGLN2-FABP5 metabolic axis therefore represents a major opportunity to improve the effects of T cell-based immunotherapies in aggressive solid tumors such as metastatic OvCa.

## MATERIALS AND METHODS

### Human specimens

Plasma samples from cancer-free women were obtained from the New York Blood Center. Malignant ascites fluid samples were obtained from patients with Stage III-IV HGSOC were procured through Surgical Pathology at Weill Cornell Medicine and Memorial Sloan-Kettering Cancer Center. All specimens were acquired with informed consent, classified as surgical discard, and kept de-identified for subsequent experimental analyses. The ascites fluid underwent initial processing by centrifugation at 4°C for 10 minutes at 1,300 rpm, with subsequent separation of supernatants from cell pellets and filtration through 0.22-μm filters to eliminate cellular debris. Processed samples were cryopreserved at −80°C in small aliquots until use. Red blood cells in cell pellets were lysed with ACK lysing buffer (Gibco). Tumor infiltrating CD8^+^ T cells (CD45^+^CD20^−^CD14^−^CD3^+^CD8^+^) were sorted from malignant ascites using a BD FACS Aria II SORP cell sorter at Flow Cytometry Core Facility in Weill Cornell Medicine. Dead cells were excluded using the DAPI. All OvCa specimens used and analyzed in this study are described in Supplementary Table 1.

### Transgenic mice and experimental OvCa models

C57BL/6J, B6.SJL-*Ptprc*^a^ *Pepc*^b^/BoyJ (CD45.1), *Eif2ak3*^fl/fl^, *Atf6*^fl/fl^, and *Vav1*^Cre^ mice were obtained from The Jackson Laboratory. *Fabp5* KO mice were provided by M. Kaczocha^50^. *Tagln2*^fl/fl^ mice where exon 3 of the *Tagln2* gene was flanked by two *loxp* sites were newly generated by the Mouse Genetics Core Facility at Memorial Sloan Kettering Cancer Center. *Ern1*^fl/fl^ and *Xbp*1^fl/fl^ mice have been previously described by our group^17,51,52^. We generated conditional-KO mice lacking PERK or ATF6 in leukocytes by crossing *Eif2ak3*^fl/fl^ or *Atf6*^fl/fl^ mice, respectively, with the *Vav1*^Cre^ strain that allows selective gene deletion in hematopoietic cells^53,54^. To generate conditional KO mice lacking TAGLN2, IRE1a or XBP1 specifically in T cells, *Tagln2* ^fl/fl^, *Ern1*^fl/fl^ or *Xbp*1^fl/fl^ mice were crossed with the *Cd4*^Cre^ strain, respectively. Female mice were housed in pathogen-free microisolator cages at the animal facilities of Memorial Sloan Kettering Cancer Center and Weill Cornell Medicine and used at 8 to 12 weeks of age for all experiments. Functional and survival experiments were conducted using age-matched, littermate controls. *In vivo* experiments included three to sixteen mice per group, based on transgenic genotype and sex availability. Mice were handled in compliance with the Institutional Animal Care and Use Committee procedures and guidelines under protocol 2011-0098.

The aggressive ID8-*Defb29*/*Vegfa* derivate was cultured and used as previously described^22,55^. The PPNM cell line (*Trp53*^−/−R172H^*Pten*^−/−^*Nf1*^−/−^*Myc*^OE^) was generously provided by Dr. R. Weinberg under MTA^45^. For tumor implantation, 1.5 × 10^6^ ID8-based ovarian cancer cells suspended in 200 μl of sterile PBS was intraperitoneally (i.p.) injected into mice. Alternatively, PPNM cells were suspended in PBS containing Matrigel (Corning Matrigel matrix, Cat# 47743-716) at 1:1 ratio, and 200 μl of the mix containing 5 × 10^5^ cells were administered i.p. into mice, as reported. Metastatic progression, ascites accumulation, and host survival were monitored over time. Tumor burden in the peritoneal cavity was assessed by live bioluminescent imaging. Briefly, PPNM-bearing mice were given a single i.p. injection of VivoGlo luciferin (2 mg/mouse. Promega, Cat# P1042) and then imaged on a Xenogen IVIS Spectrum In Vivo imaging system at the Weill Cornell Research Animal Resource Center. All cell lines were verified for mycoplasma contamination and maintained under prophylactic plasmocin supplementation (Invivogen, Cat# ant-mpt).

### RNA isolation and real time quantitative PCR (RT-qPCR) analysis

Total RNA was isolated using the QIAzol lysis reagent (Qiagen) according to the manufacturer’s instructions. RNA (0.1–1 μg) was reverse-transcribed to generate cDNA using the qScript cDNA synthesis kit (Quantabio). Quantitative RT-PCR was performed using PerfeCTa SYBR green fastmix (Quantabio) on a QuantStudio 6 Flex real-time PCR system (Applied Biosystems). Normalized gene expression was calculated by the comparative threshold cycle method using *ACTB* for human or *Actb* for mouse as endogenous controls. All primers used in this study are described in Supplementary Table 3.

### T cell siRNA and mRNA electroporation

T cells were transfected with either small interfering RNA (siRNA) or messenger RNA (mRNA) via electroporation utilizing the Neon Transfection System (Thermo Fisher Scientific). The following siRNAs and mRNAs were used: siRNA-ON-TARGETplus Non-targeting Control Pool (Horizon), ON-TARGETplus Mouse *Fabp5* siRNA (Horizon). mRNA-CleanCap^®^ Ovalbumin mRNA (5-methoxyuridine; L-7210) was obtained from TriLink Biotehcnology. Mouse *Tagln2* and human *FABP5* mRNAs were designed and synthesized by TriLink Biotechnology and Genescript, respectively. Briefly, naïve splenic CD8^+^ T cells were isolated and stimulated with plate-bound anti-CD3ε (145-2C11, 5 μg/ml) and soluble anti-CD28 (37.51, 1 μg/ml; BD Pharmingen) antibodies for 24 hours. On the following day, activated viable T cells were counted and resuspended in Neon Buffer T, then mixed with indicated siRNA or mRNA. The mixture was pipetted using the Neon pipette and tips and plugged the Neon pipette into position in the Neon transfection device. Electroporation was performed at 3 pulses, 10ms pulse width, 1600V. T cells were subsequently expanded on plate-bound anti-CD3ε and soluble anti-CD28 antibodies in complete medium with 100 units per mL of IL-2 (200-02; PeproTech) for additional 2 days.

### Flow cytometry

Flow cytometry was conducted using fluorochrome-conjugated antibodies purchased from BioLegend, unless stated otherwise. Cells were washed with PBS, Fc-gamma receptor-blocked using TruStain fcX^™^ (anti-mouse CD16/32, 93), LIVE/DEAD^™^ Fixable Near-IR dead cell stain for live/dead discrimination (Invitrogen) and then stained for surface or intracellular markers at 4°C in the dark for 30 minutes. For staining of mouse cells we used: anti-CD45 (30-F11, 1:200), anti-CD3 (17A2, 1:200), anti-CD4 (RM4-5, 1:200), anti-CD8α (53-6.7, 1:200), anti-CD44 (IM7, 1:200), anti-CD62L (MEL-14, 1:200) anti-CD11c (N418, 1:200), anti-I-A/I-E (M5/114.15.2, 1:200; Tonbo biosciences), anti-CD11b (M1/70, 1:200), anti-F4/80 (BM8, 1:200), anti-NK1.1 (PK136, 1:200), anti-CD19 (6D5, 1:200), CD45.1 (A20, 1:200), CD45.2 (104, 1:200; BD Pharmingen), anti-FABP5 (primary; AF1476, 1:100; R&D Systems, secondary; Donkey anti-Goat IgG (H+L) Alexa Fluor^™^ Plus 647; A32849, 1:200; Invitrogen), anti-Transgelin-2 (primary; 15508-1-AP, 1:100; Thermo Fisher Scientific, secondary; Alexa Fluor^™^ 488 goat anti-rabbit IgG (H+L); A11008, 1:200; Invitrogen; R-phycoerythrin goat anti-rabbit IgG (H+L); P2771MP, 1:200; Invitrogen), anti-Ki-67 (16A8, 1:50), anti-IFNγ (XMG1.2, 1:100), anti-TNFα (MP6-XT22, 1:100), anti-GranzymeB (QA16A02, 1:100). To stain human cells we used: anti-CD45 (HI30, 1:200), anti-CD3 (HIT3a, 1:200), anti-CD8a (HIT8a, 1:200), anti-CD19 (HIB19, 1:200), anti-CD45RO (UCHL1, 1:200), anti-CCR7 (150503, 1:200; BD Pharmingen), anti-CD44 (515, 1:200; BD Pharmingen), anti-XBP1s (Q3-695, 1:50; BD Pharmingen), anti-FABP5 (primary; AF1476, 1:100; R&D Systems, secondary; Donkey anti-Goat IgG (H+L) Alexa Fluor^™^ Plus 647; A32849, 1:200; Invitrogen), anti-Transgelin-2 (primary; 15508-1-AP, 1:100; Thermo Fisher Scientific, secondary; Alexa Fluor^™^ 488 goat anti-rabbit IgG (H+L); A11008, 1:200; Invitrogen). Staining of transcription factor XBP1s, intracellular proteins (Ki-67, TAGLN2 and Total FABP5) and cytokines (IFNγ, TNFα and GranzymeB) was carried out using Foxp3/transcription factor staining buffer set (eBioscience) according to the manufacturer’s instructions. For in vitro lipid uptake experiments, 2 × 10^5^ human or mouse CD8^+^ T cells in complete medium containing 1 μmol/L BODIPY^™^ 500/510 C1, C12 (D3823; Thermo Fisher Scientific) for 30 minutes at 37 °C with 5% CO2. To measure the mitochondrial mass and membrane potential, WT or Tagln2 KO CD8^+^ T cells pre-activated for twenty-four hours in complete medium were cultured in complete medium or glucose-free medium with or without oleic acids for forty-eight hours, respectively. Then, cells were stained with 10 nM MitoTracker Deep Red (M46753; Thermo Fisher Scientific) and 100 nM MitoTracker Green (M46750; Thermo Fisher Scientific) for 15 min at 37 °C with 5% CO2 followed by Live/Dead and cell surface staining. Flow cytometry was performed on a LSRII or a Fortessa-X20 instruments (BD Biosciences) and data were analyzed using FlowJo v.10 (TreeStar).

### Immunoprecipitation and LC-MS/MS analysis

For LC-MS/MS analysis of FABP5 interacting proteins in CD8^+^ T cells, activated mouse CD8^+^ T cell pellets were lysed using Pierce^™^ IP Lysis Buffer (Thermo Fisher Scientific, Cat# 87787) supplemented with a protease and phosphatase inhibitor tablet (Millipore, Cat# 11697498001 and Roche, Cat# 04906837001) for 30 min at 4°C. Homogenates were centrifuged at 15,000 rpm. for 10 min at 4 °C, and the supernatants were collected. Protein concentrations were determined using a BCA protein assay kit (Thermo Fisher Scientific, Cat# 23225). Lysates were then incubated for 3 hours with Dynabeads^™^ MyOne^™^ Streptavidin T1 (65601; Invitrogen) under continuous rotation to remove non-specific proteins. After bead removal, goat IgG biotinylated control (BAF108; R&D systems) or goat anti-mouse FABP5 biotinylated antibodies (BAF1476; R&D Systems) was added for overnight incubation at 4°C under continuous rotation, and then incubation with Dynabeads^™^ MyOne^™^ Streptavidin T1 was carried out for 2 hours at 4°C under continuous rotation to capture immune complexes. After wash by PBS containing 0.05% tween-20, immune complexes were eluted from the magnetic beads with 0.1M glycine∙HCl (pH2.02) with mixing for 10 min, followed by neutralized with 1M Tris (pH 8.5). IgG or FABP5 pull-down elution sample was fractionated on 4–12% Bis-Tris gels (Invitrogen) and running gels were stained with SimplyBlue^™^ SafeStain Coomassie (LC6060; Invitrogen) mass spectrometry analyses, which were performed by the Proteomics and Lipidomics Core Facility of Weill Cornell Medicine following standard methods.

### Co-immunoprecipitation assays

To validate the interaction between FABP5 and TAGLN2, activated mouse CD8^+^ T cell pellets were lysed using Pierce^™^ IP Lysis Buffer (Thermo Fisher Scientific, Cat# 87787) supplemented with a protease and phosphatase inhibitor tablet (Millipore, Cat# 11697498001 and Roche, Cat# 04906837001) for 30 min at 4°C. Homogenates were centrifuged at 15,000 rpm. for 30 min at 4 °C, and the supernatants were collected. Protein concentrations were determined using a BCA protein assay kit (Thermo Fisher Scientific, Cat# 23225). Lysates were precleared by adding Protein A/G magnetic beads (88803; Thermo Fisher Scientific) for 1 hour at 4°C under continuous rotation. After bead removal, goat IgG isotype control (02–6202; Invitrogen) or goat anti-mouse FABP5 (AF1476; R&D Systems) antibodies for FABP5 immunoprecipitation experiment or rabbit IgG isotype control (026102; Invitrogen) or rabbit anti-mouse Transgelin-2 (15508-1-AP; Thermo Fisher Scientific) for TAGLN2 immunoprecipitation experiment was added for overnight incubation at 4°C under continuous rotation, and then incubation with Protein A/G magnetic beads (88803; Thermo Fisher Scientific) was carried out for 1 hours at room temperature under continuous rotation to capture immune complexes. After wash by 1X TBS containing 0.05% tween-20 and 0.5M NaCl, beads were removed by magnetic. Each precipitated sample was separated via SDS-PAGE and transferred onto PVDF membranes following standard protocols. The following antibodies were used for western blotting: anti-FABP5 (AF1476) (R&D Systems), anti-Transgelin-2 (15508-1-AP) (Thermo Fisher Scientific), Rabbit anti-Goat IgG Fc secondary antibody conjugated with HRP (Thermo Fisher Scientific, Cat# 31433), Goat anti-Rabbit IgG Fc secondary antibody conjugated with HRP (Thermo Fisher Scientific, Cat# A16116). SuperSignal West Pico (Thermo Fisher Scientific, Cat# 34580) or Femto chemiluminescent substrates (Thermo Fisher Scientific, Cat# 34095) were used to image blots in an iBright CL1000 instrument (Thermo Fisher Scientific).

### Cytokine quantification

Human undiluted ascites samples were submitted to Eve Technologies^™^ Assay Services for analysis using the Human Cytokine/Chemokine 71-Plex Discovery Assay^®^ Array.

### Plasmid constructs and luciferase reporter assays

Expression constructs used for luciferase-based assays are XBP-1 2: pFLAG.XBP1p.CMV2 (Addgene plasmid # 21833) and Flag-p50(1–435) (Addgene plasmid # 44747) while reporter construct used is pGL3-Tagln2 promoter (−738 to +134). For dual luciferase reporter assays, 2 × 10^4^ HEK-293T cells were plated overnight in flat bottom 96-well plates and transfected with the indicated plasmids using jetPRIME (Polyplus-transfection) according to the manufacturer’s protocol. Briefly, 18ng of reporter and 2 ng of Renilla plasmid were co-transfected with various ratios (wt:wt) of expression plasmids (reporter: expression plasmid = 1:1, 1:2 or 1:3) and pcDNA3.1, which was added to reach a total 200ng of plasmid per well. After forty-eight hours, cells were washed with PBS and lysed Passive Lysis Buffer according to the manufacturer’s protocol (Dual Luciferase Reporter Assay System, #E1960; Promega). Firefly and Renilla luciferase activities were measured in white-bottom 96-well plates using an automated luminometer (SpectraMax iD3 Multi-Mode Microplate Reader; Molecular Devices). The reporter activity (Firefly) was normalized to its own Renilla luciferase activity.

### Chromatin immunoprecipitation and PCR (ChIP-PCR)

Pre-activated mouse CD8^+^ T cells were incubated in complete medium in the presence or absence of tunicamycin (1 μg/ml) for 16 hours. Cells were then washed and fixed in 1% formaldehyde for ChIP assay. Cross-linking was terminated using 0.125M glycine. Nuclear extracts were collected and resuspended in a lysis buffer containing a high salt concentration. Chromatin sonication was carried out using a cell disruptor (Branson 150D Sonifier 150). The chromatin solution was precleared by adding Protein A/G magnetic beads (88803; Thermo Fisher Scientific) for 1 hour at 4°C under continuous rotation. After bead removal, anti-mouse IgG1 (MOPC-21; Biolegend) or anti-mouse XBP1s (9D11A43; Biolegend) antibodies was added for overnight incubation at 4°C under continuous rotation, and then incubation with Protein A/G magnetic beads was carried out for 2 hours at 4°C under continuous rotation. Beads were removed by magnetic and sequentially washed with lysis buffer high salt, wash buffer and elution buffer. Cross-links were reversed by heating at 65°C in a water bath, and the DNA bound to the beads isolated by extraction with phenol/chloroform/isoamylalcohol. Within the regions of interest, XBP1s binding is represented as the percentage of enrichment over input, and calculation was conducted using the change-in-threshold (2^−ΔΔCT^) method. The sequences for primer pairs used for ChIP-quantitative PCR analyses are mentioned in Supplementary Table 3.

### Immunofluorescence and confocal microscopy

Naive or activated CD8^+^ T cells in the absence or presence of tunicamycin (2.5 – 3 × 10^6^) were transferred onto poly-L-lysine pre-coated glass coverslips (Neuvitro) and incubated at 37 °C with 5% CO2 for 30 min. The coverslips were washed with cold PBS three times between each step. Cells were subsequently fixed with ice-cold acetone for 5 min at room temperature, warmed 2% formaldehyde for 20 min at room temperature and ice-cold 70% ethanol for 5 min at 4°C. Then, cells were permeabilized for 20 min in PBS containing 0.5% Triton X-100 and 10% FBS at room temperature. Then, the coverslips were blocked for 1 h in PBS containing 10% BSA at room temperature, followed by incubation at 4 °C overnight with primary antibodies rabbit anti-Transgelin-2 (15508-1-AP, Thermo Fisher Scientific, 1:100) in PBS containing 0.1% tween 20 and 1% BSA, protected from light. Then, secondary antibodies Alexa Fluor 488-conjugated goat anti-rabbit IgG (Molecular Probes, 1:1000) were added for 1 h at room temperature in the dark. Cells were counterstained with 4′,6-diamidino-2-phenylindole (DAPI, Thermo Fisher Scientific, 0.5 μg/ml) for 5 min at room temperature in the dark. After washing and removing excess solution, the flipped coverslips were placed on the mounting medium (Southern Biotechnology). Slides were allowed to dry in the dark for 1 h in a humid chamber at room temperature. Slides were then sealed with fingernail polish before examination. Digital confocal images were captured on a Zeiss LSM 880 Confocal Microscope with the Airy Scan high-resolution detector at the Weill Cornell CLC Imaging Core Facility.

### Single-cell RNA-sequencing

#### Mouse:

The total CD3^+^ T cells (DAPI^−^CD45^+^CD11b^−^CD19^−^NK1.1^−^MHCll^−^CD3^+^) of peritoneal lavage were isolated from mice bearing ID8-*Defb29/Vegfa* ovarian cancer for 23 days and subjected to single-cell RNA sequencing (scRNA-seq). Library preparation, sequencing, and raw data preprocessing were performed at the Genomics Resources Core Facility of Weill Cornell Medicine. Raw gene expression matrices were generated for each sample by the Cell Ranger (v.3.0.2) Pipeline coupled with mouse reference version GRCm38 (mm10). The output-filtered gene expression matrices were analyzed by R software (v.4.2.2) with the Seurat package^56^ (v.3.0.0). In brief, genes expressed at a proportion >0.1% of the data and cells with >200 genes detected were selected for further analyses. Low-quality cells were removed if they met the following criteria: <800 unique molecular identifiers (UMI), <500 genes, or >5% UMIs derived from the mitochondrial genome. Further, gene expression matrices were normalized by the Normalize-Data function, and 2,000 features with high cell-to-cell variation were calculated using the FindVariableFeatures() function. To reduce the dimensionality of the data sets, the RunPCA() function was conducted with default parameters on linear transformation–scaled data generated by the ScaleData() function. In the end, we clustered cells using the FindNeighbors() and FindClusters() functions and performed nonlinear dimensional reduction with the RunUMAP() function with default settings. All details regarding the Seurat analyses performed in this work can be found in the website tutorial (https://satijalab.org/seurat/v3.0/pbmc3k_tutorial.html). Data were deposited under NCBI Gene Expression Omnibus (GEO) Accession number GSE248595.

#### Human:

For the analysis of scRNA-seq data of tumor-infiltrating lymphocytes (TILs) in HGSOC patients, preprocessed counts from 11 HGSOC treatment-naïve tumor specimens^46^ were downloaded from Gene Expression Omnibus (GEO) with accession code GSE165897. Deeper annotation of T cell subtypes was obtained by projecting the CD4^+^ and CD8^+^ T cells to a reference atlas using ProjecTILs^57^. Then, differential expression analysis was performed within each subtype comparing cells with and without TALGN2 expression using *FindMarkers* function from Seurat package^56^ (v4.3.0). From this comparison, the fold-change (in logarithmic scale) was used in a GSEA analysis to test the enrichment of ER stress gene signature (Supplementary Table 2) in Tagln2^hi^ CD8^+^ T_EM_ TILs using clusterProfiler package^58^ (v4.5.0).

### Seahorse assays

Purified naïve CD8^+^ T cells from wild type mice were activated with CD3ε and CD28 (Purified NA/LE Hamster anti-mouse CD3ε (145-2C11) and anti-mouse CD28 (37.51), BD pharmingen). Pre-activated CD8^+^ T cells were then stimulated with or without Tunicamycin at various concentrations for 16 hours at 37 °C with 5% CO2. In some experiments, pre-activated CD8^+^ T cells were overexpressed with mRNAs encoding control ovalbumin (*Ctrl*-mRNA) or mouse TAGLN2 (*Tagln2*-mRNA). After treatment, cells were washed and resuspended in nonbuffered RPMI 1640, pH 7.4 (Agilent) supplemented with 10 mM glucose, 2 mM L-glutamine, and 1 mM pyruvate. 2.5 × 10^5^ cells per well were plated onto poly-D-lysine precoated XF96 cell culture microplates (Agilent Technologies). The oxygen consumption rate (OCR) and extracellular acidification rate (ECAR) were measured on an XFe96 extracellular flux analyzer (Agilent Technologies). After basal OCR and ECAR measurements were obtained, an OCR trace was recorded in response to oligomycin (1 μM), carbonyl cyanide-p-(trifluoromethoxy) phenylhydrazone (FCCP, 1 μM), and rotenone and antimycin (0.5 μM each) following the XF Cell Mito Stress test kit (Agilent).

To evaluate the contribution of fatty acids as mitochondrial fuel usage, pre-activated CD8^+^ T cells stimulated with Tunicamycin either overexpressed with mRNAs encoding control ovalbumin (*Ctrl-*mRNA) or mouse TAGLN2 (*Tagln2*-mRNA) were seeded in the Seahorse medium containing 10 mM glucose, 2 mM L-glutamine, and 1 mM pyruvate. After recording basal OCR and ECAR measurements, cells were injected with corresponding base medium (control), or etomoxir (4 μM, Tocris Bioscience) followed by oligomycin, FCCP, and rotenone and antimycin injection using the XF standard substrate oxidation test (Agilent). Metabolic parameters were calculated as follows: basal respiration = last rate measurement before oligomycin injection – minimum rate measurement after rotenone and antimycin injection; maximal respiration = maximum rate measurement after FCCP injection – minimum rate measurement after rotenone and antimycin injection. At least five technical replicates per each sample were examined. After analysis, the cell numbers of each well were determined by nuclear DNA staining with Hoechst 33342 (Sigma), and OCR and ECAR values were normalized accordingly.

### Western blotting

PPNM cancer cells were washed twice in 1× cold PBS and cell pellets were lysed using RIPA lysis and extraction buffer (Thermo Fisher Scientific, Cat# 89900) supplemented with a protease and phosphatase inhibitor tablet (Millipore, Cat# 11697498001 and Roche, Cat# 04906837001). Homogenates were centrifuged at 14,000 rpm. for 30 min at 4 °C, and the supernatants were collected. Protein concentrations were determined using a BCA protein assay kit (Thermo Fisher Scientific, Cat# 23225). Equivalent amounts of protein were separated by SDS–PAGE and transferred onto PVDF membranes following the standard protocol. The following antibodies were used: anti-FSHR (PA5-95380; Thermo Fisher Scientific), HRP-Conjugated Beta Actin Monoclonal Antibody (Thermo Fisher Scientific, Cat# MA5-15739-HRP), Goat anti-Rabbit secondary antibody conjugated with HRP (Thermo Fisher Scientific, Cat# 32460). SuperSignal West Pico (Thermo Fisher Scientific, Cat# 34580) was used to image blots in an iBright CL1000 instrument (Thermo Fisher Scientific).

### Plasmids and retroviral transduction

pBMN-I-GFP and pBMN-I-GFP-FSHCER retroviral vectors were kindly provided by J.R. Conejo-Garcia^49^. Tagln2 cDNA was subcloned with T2A sequences in pBMN-I-GFP-FSHCER (pBMN-I-GFP-FSHCER-2A-Tagln2) vector by Genescript Biotech. Retroviruses were produced by transfecting 6 μg of the retroviral expression vector together with 4 μg of the retroviral packaging vector (pCL-Eco, Addgene plasmid # 12371) into retroviral packaging cell line Platinum-E (RV-101, Cell Biolabs, Inc.), using Lipofectamine^™^ 3000 Transfection Reagent (Thermo Fisher Scientific). Forty-eight hours after transfection, high-titer viral supernatant was collected. For transduction, 2 × 10^6^ cells/mL of naïve CD8^+^ T cells or Pan T cells from B6.SJL-*Ptprc*^a^ *Pepc*^b^/BoyJ (CD45.1) mice were isolated and activated with plate-bound anti-CD3 and soluble anti-CD28 overnight, followed by spin-infection with viral supernatant for 90 minutes at 2500 rpm, 30°C in the presence of 8 μg/mL of polybrene. Every 2 days, replenish fresh complete media with added 100 units per mL of IL-2 (200-02; PeproTech) to expand appropriate number of cells for further functional experiments. At day 7, transduced T cells were used for in vitro killing assays or adoptive cell transfer in PPNM ovarian tumor-bearing mice.

### In vitro killing assay

PPNM OvCa cells were plated at a density of 1 × 10^4^ cells per well in flat bottom 96-well plates containing 200 μL of culture medium. These plates were then incubated overnight at 37 °C with 5% CO2. On the following day, tumor conditioned medium was washed away and added fresh complete medium with no beta-mercaptoethanol and the same number of Mock- or CER-transduced CD8^+^GFP^+^ T cells per well (in 200 μL). Following eighteen hours, CD8^+^ T cells and PPNM cells were collected by trypsinization and proceeded to flow cytometric analysis of cellular cytotoxicity (target cells - CD45^−^CD3^−^ gated PPNM ovarian cancer cells) by using Annexin V Apoptosis Detection Kit with PI (Biolegend).

### Tumor implantation and CER T cell treatments

Wild type C57BL/6J (CD45.2) female mice were implanted via intraperitoneal (i.p.) injection with 5 × 10^5^ PPNM OvCa cells suspended in PBS containing Matrigel (Corning Matrigel matrix, Cat# 47743-716) at 1:1 ratio (200 μL per mouse). After 7 days, tumor-bearing mice were randomized into treatment groups (*n* = 16 each). On days 8 and 15, mice were treated i.p. with 1 × 10^7^ cells per mouse, either CER or CER-Tagln2 T cells, which were generated from B6.SJL-Ptprca Pepcb/BoyJ (CD45.1) mice. Metastatic progression, ascites accumulation, and host survival were monitored over time. Tumor burden in the peritoneal cavity was assessed by live bioluminescent imaging.

### Statistical analyses

All statistical analyses were performed using GraphPad Prism 10 software. Significance for pairwise correlation analysis was calculated using the Spearman’s correlation coefficient (r). Comparisons between two groups were assessed using unpaired or paired (for matched comparisons) two-tailed Student’s t-test, or non-parametric Mann–Whitney U-test. Multiple comparisons were assessed by one-way ANOVA, including Tukey’s multiple comparisons tests. Survival rates were compared using the log-rank test. Data are presented as mean ± s.e.m. Exact *p*-values are shown, and *p*-values of <0.05 were considered to be statistically significant.

## Extended Data

## Supplementary Material

Supplement 1

## Data Availability

The data that support the findings of this study are included in the text and in the Supplementary Information. Additional datasets used in the study are: scRNA-seq for infiltrating lymphocytes from human metastatic melanoma (GSE72056) and bulk-RNA seq for CD8^+^ TILs from a mouse autochthonous liver tumor model (GSE126974). *TAGLN2* transcript data in the indicated different CD8^+^ T cell populations from human peripheral blood mononuclear cells (PBMC) was downloaded from The Human Protein Atlas database (https://www.proteinatlas.org/ENSG00000158710-TAGLN2/immune+cell). Mouse single-cell RNA sequencing (scRNA-seq) data were deposited under NCBI GEO Accession number (GSE248595). For the analysis of human scRNA-seq data, preprocessed counts from 11 HGSOC treatment-naive specimens (Ref. 46) were downloaded from Gene Expression Omnibus (GEO) with accession code GSE165897. All other data supporting the findings of this study are available from the corresponding author on reasonable request. Source data are provided with this paper.
