# Supplementary material for: Transgelin 2 guards T cell lipid metabolic programming and anti-tumor function
Source: Res Sq. 2023 Dec 14:rs.3.rs-3683989. Preprint. [Version 1] doi: 10.21203/rs.3.rs-3683989/v1 (PMC10760247; doi:10.21203/rs.3.rs-3683989/v1)
Supplement: Supplement 1 [file NIHPPrs3683989v1-supplement-1.pdf]

Supplementary Information

Transgelin 2 guards T cell lipid metabolic programming and anti-tumor function

Sung-Min Hwang[1,2], Deepika Awasthi[1,2], Jieun Jeong[3], Tito A. Sandoval[1,2], Chang-Suk Chae[1,2,‡], Yusibeska Ramos[1], Chen Tan[1,2], Matías Marin Falco[4], Ian McBain[5], Bikash Mishra[5,6], Lionel B. Ivashkiv[5,6], Dmitriy Zamarin[7], Evelyn Cantillo[1,2], Eloise Chapman-Davis[1,2], Kevin Holcomb[1,2], Diana K. Morales[1], Paulo C. Rodriguez[8], Jose R. Conejo-Garcia[9,10], Martin Kaczocha[11,12,13], Anna Vähärautio[4,14], Minkyung Song[1,2,#] and Juan R. Cubillos-Ruiz[1,2,5,†]

Affiliations:

[1] Department of Obstetrics and Gynecology, Weill Cornell Medicine. New York, NY 10065, USA.
[2] Sandra and Edward Meyer Cancer Center, Weill Cornell Medicine. New York, NY 10065, USA.
[3] Cancer Biology and Genetics Program, Memorial Sloan-Kettering Cancer Center, New York, NY 10065, USA.
[4] Research Program in Systems Oncology, Research Programs Unit, Faculty of Medicine, University of Helsinki, Helsinki, Finland.
[5] Weill Cornell Graduate School of Medical Sciences. New York, NY 10065. USA.
[6] HSS Research Institute and David Z. Rosensweig Genomics Research Center, Hospital for Special Surgery, New York, NY, USA.
[7] Tisch Cancer Institute, Icahn School of Medicine at Mount Sinai, New York, NY 10029, USA.
[8] Department of Immunology, H. Lee Moffitt Cancer Center & Research Institute. Tampa, FL, USA.
[9] Department of Integrated Immunobiology, Duke School of Medicine, Durham, NC 27710, USA.
[10] Duke Cancer Institute, Duke School of Medicine, Durham, NC 27710, USA.
[11] Department of Anesthesiology, Renaissance School of Medicine, Stony Brook University, Stony Brook, NY, USA.
[12] Institute of Chemical Biology and Drug Discovery, Stony Brook University, Stony Brook, NY, USA.
[13] Stony Brook University Pain and Analgesia Research Center (SPARC), Renaissance School of Medicine, Stony Brook University, Stony Brook, NY, USA.
[14] Foundation for the Finnish Cancer Institute, Helsinki, Finland.
[‡] Present address: Research Institute, National Cancer Center, 323 Ilsan-ro, Goyang, Gyeonggi-Do, 10408, Republic of Korea.
[#] Present address: Departments of Integrative Biotechnology and of Biopharmaceutical Convergence, Sungkyunkwan University. Suwon, Gyeonggi-do, Republic of Korea.

[†] **Correspondence:** Juan R. Cubillos-Ruiz, Ph.D.
Associate Professor of Microbiology and Immunology
Well Cornell Medicine, New York, NY
Email: jur2016@med.cornell.edu

Supplementary Information includes:

Supplementary Table 1. Information on human HGSOC patient-derived specimens.

Supplementary Table 2. List of genes in the ER stress response signature used for GSEA analyses.

Supplementary Table 3. Primer Sequences

Supplementary Figure 1: Gating strategy used for FACS analyses, related to Fig.1 and Fig. 3.

Supplementary Figure 2: Original western blot pictures

Supplementary Table 1. Information on human HGSOC patient-derived specimens.

Sample type	Patient ID	Purpose of experiment	Patient information
Ascites	A28	In vitro analyses (Cell-free supernatants)	Primary OvCa or peritoneal cancer; Chemo-naive
	A29		Primary OvCa; Chemo-naive
	A30		Metastatic OvCa; Primary surgical debulking; Chemo-naive
	A35		Primary OvCa; Chemo-naive
	A43		Primary OvCa; Chemo-naive
	A20	TIL analyses	High-grade serous OvCa; Chemo-naive
	A21		Undetermined
	A22		Recurrent OvCa Stage 1C
	A25		Recurrent OvCa
	A26		Recurrent peritoneal OvCa; Multi-drug resistant; Several times of Chemotherapies
	A28		Primary OvCa or peritoneal cancer; Chemo-naive
	A30		High-grade serous OvCa; Chemo-naive
	A33		High-grade serous endometrial or ovarian carcinoma
	A35		High-grade serous OvCa; Chemo-naive
	A36		High-grade serous OvCa; Chemo-naive
	A38		High-grade serous OvCa; Chemo-naive
	A39		Mucinous OvCa, Stage lll; Chemo-naive
	A40		Adenocarcinoma; cytology high grade; Mullerian origin (includes ovarian and endometrium)
	A41		Undetermined
	A46		Recurrent Fallopian tube cancer; Neoadjuvant chemotherapy
	A3	Sort $CD45^+CD3^+CD8^+$ TILs for qRT-PCR analyses	High-grade serous OvCa
	A6		High-grade serous OvCa
	A7		High-grade serous OvCa
	A9		High-grade serous OvCa
	A14		High-grade serous OvCa; platinum-resistant, Recurrent peritoneal OvCa
	A15		High-grade serous OvCa
	A16		High-grade serous OvCa; terminal stage
	A17		High-grade serous OvCa; Chemo-naive
	A18		High-grade serous OvCa; Chemo-naive
	A19		High-grade serous OvCa; Chemo-naive
	A28		Primary OvCa or peritoneal cancer; Chemo-naive
	A29		Primary OvCa; Chemo-naive
	A30		High-grade serous OvCa; Chemo-naive
	A35		High-grade serous OvCa; Chemo-naive
	A36		High-grade serous OvCa; Chemo-naive
	A38		High-grade serous OvCa; Chemo-naive

Supplementary Table 2. Genes in the ER stress signature used for GSEA analyses.

GENE SYMBOL	GENE NAME
Hyou1	hypoxia up-regulated 1
Hspa14	heat shock protein 14
Sec61a1	SEC61 translocon subunit alpha 1
Sec24d	SEC24 homolog D, COPII coat complex component
Hspa13	heat shock protein 70 family, member 13
Sec24c	SEC24 homolog C, COPII coat complex component
Ambra1	autophagy/beclin 1 regulator 1
Surf4	surfeit gene 4
Atg13	autophagy related 13
Xbp1	X-box binding protein 1
P4hb	prolyl 4-hydroxylase, beta polypeptide
Fam129a	family with sequence similarity 129 member A
Pdia4	protein disulfide isomerase associated 4
Spcs3	signal peptidase complex subunit 3 homolog (S. cerevisiae)
Surf6	surfeit gene 6
Atf6	activating transcription factor 6
Dapk1	death associated protein kinase 1
Dnajb9	DnaJ heat shock protein family (Hsp40) member B9
Dnajc3	DnaJ heat shock protein family (Hsp40) member C3
Mfn2	mitofusin 2
Pdia6	protein disulfide isomerase associated 6
Ccdc47	coiled-coil domain containing 47
Dnajc14	DnaJ heat shock protein family (Hsp40) member C14
Sil1	SIL1 nucleotide exchange factor
Sec16a	SEC16 homolog A, endoplasmic reticulum export factor
Tmx3	thioredoxin-related transmembrane protein 3
Sec23b	SEC23 homolog B, COPII coat complex component
SEC31A	SEC31 homolog A, COPII coat complex component
Gosr2	golgi SNAP receptor complex member 2
Asns	asparagine synthetase
Atf4	activating transcription factor 4
Ddit3	DNA-damage inducible transcript 3
Ddit4	DNA-damage inducible transcript 4
Edem1	ER degradation enhancer, mannosidase alpha-like 1
Hspa5	heat shock protein 5

Supplementary Table 3. Primer Sequences.

Name	Forward Primer (5'-3')	Reverse Primer (5'-3')
**Primers for RT-qPCR (Human)**		
ACTB	GCGAGAAGATGACCCAGATC	CCAGTGGTACGGCCAGAGG
TAGLN2	ATGGCACGGTGCTATGTGAG	CCCACCCAGATTCATCAGCG
XBP1s	CTGAGTCCGCAGCAGGTG	TCCAAGTTGTCCAGAATGCC
FABP5	TGAAGGAGCTAGGAGTGGGAA	TGCACCATCTGTAAAGTTGCAG
CD36	AAGCCAGGTATTGCAGTTCTTT	GCATTTGCTGATGTCTAGCACA
FABP4	ACTGGGCCAGGAATTTGACG	CTCGTGGAAGTGACGCCTT
IFNG	TCGGTAACTGACTTGAATGTCCA	TCGCTTCCCTGTTTTAGCTGC
TNFA	GGAGAAGGGTGACCGACTCA	CTGCCCAGACTCGGCAA
GZMB	CCCTGGGAAAACACTCACACA	GCACAACTCAATGGTACTGTCG
**Primers for RT-qPCR (Mouse)**		
Actb	CTCAGGAGGAGCAATGATCTTGAT	TACCACCATGTACCCAGGCA
Tagln2_Exon3	CTTGAGGCTCACCACAGGAA	TTGAAGGCCATCGAAGAGGC
Tagln2	TCTTTGCCATCACCACAGCTGCTCAGAATG	CGTGCCGTCCTTGAGCCACTTCTGGAAGTT
Xbp1s	AAGAACACGCTTGGGAATGG	CTGCACCTGCTGCGGAC
Sec61a1	CTATTTCCAGGGCTTCCGAGT	AGGTGTTGTACTGGCCTCGGT
Dnajb9/Erdj4	TAAAAGCCCTGATGCTGAAGC	TCCGACTATTGGCATCCGA
**Primers for luciferase reporter construct generation**		
Tagln2-promoter	GGGGTACCCCCACCCCTCAACTATTGCT	CCGCTCGAGCGGCGTCCAAGAGGGCTGG
**Primers for ChIP-qPCR**		
XBP1s non-binding site (NB)	AGGGGTAGAAAAGTGCCTGC	CAGTGAGGTCACTCCTTGCC
XBP1s binding site 1 (BS1)	AGTTAAATGGCAAGCAGAACCAC	GCGCCTTCCTACAGGATAGAGTA
XBP1s binding site 2 (BS2)	TTCCTGCCTACTGACCACCT	GCAAGGGCCAAGAGGGTTTA

Supplementary Figure 1

a

Gating strategy used in flow cytomentry analysis experiments [Fig1 and 3 - Human]

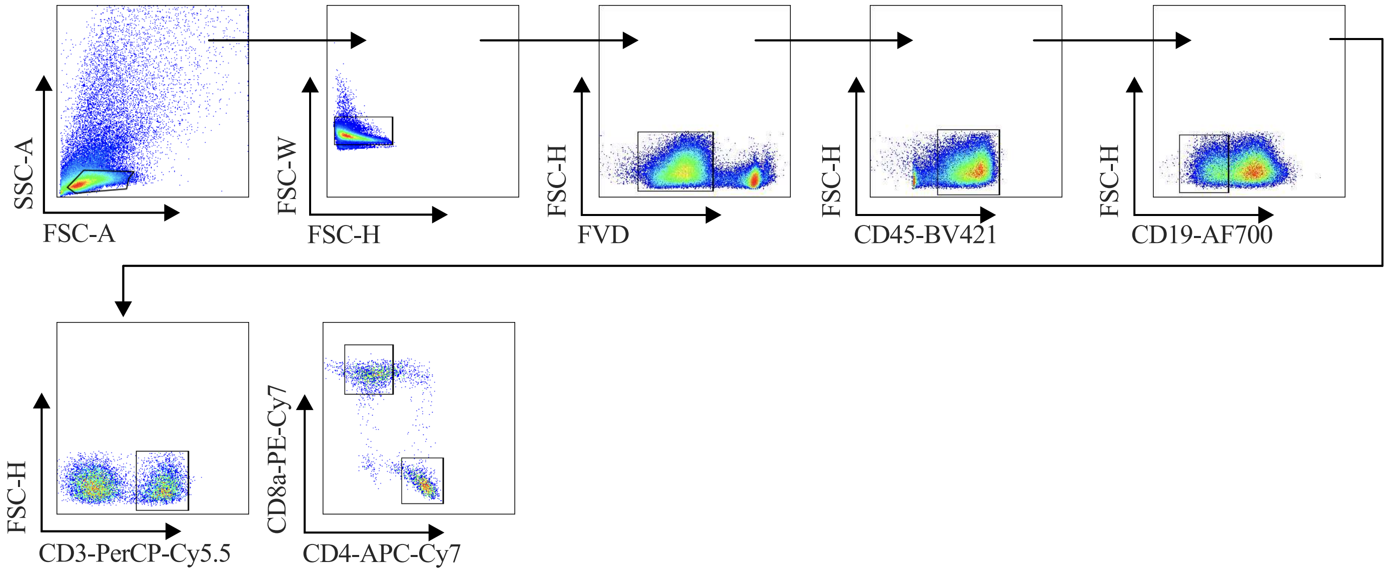

b

Gating strategy used in flow cytomentry analysis experiments [Fig1, 3 and 4 - Mouse]

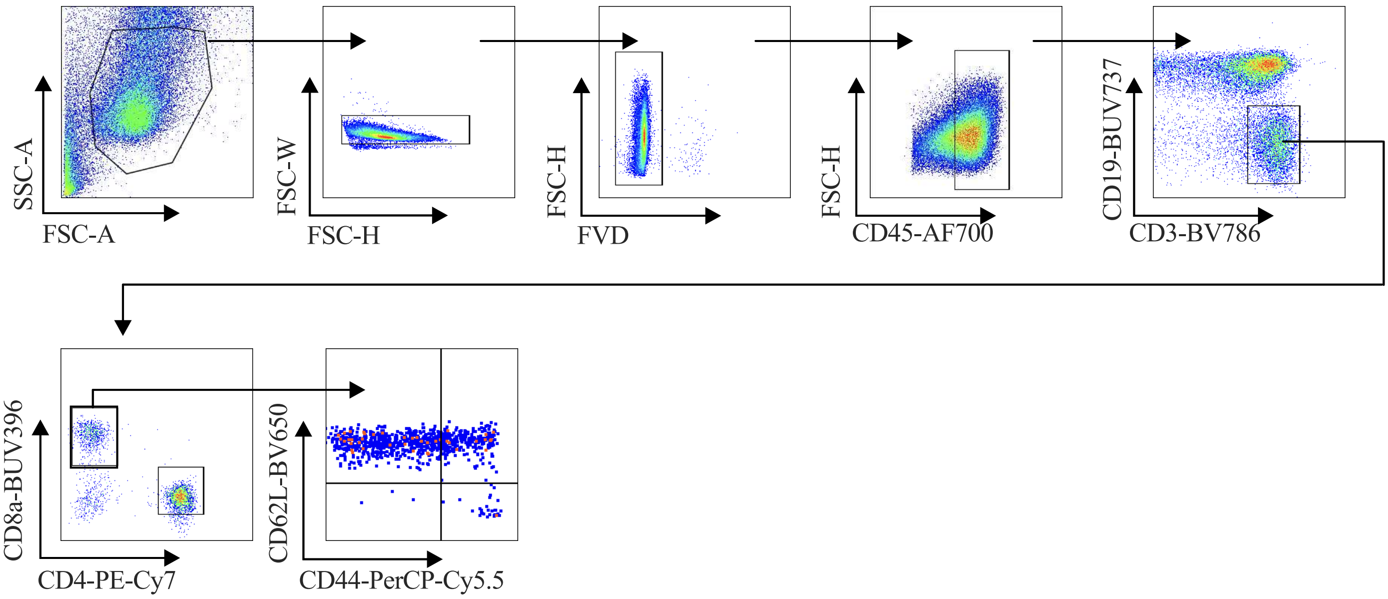

c

Representative purity of naive CD8^{+} T cells after negative magnetic isolation from human PBMCs or mouse lymphocytes from spleens and lymph nodes
[Fig1, 2, 4 and 5/ Extended Data Fig.1, 2, 4, 7 - Human/Mouse]

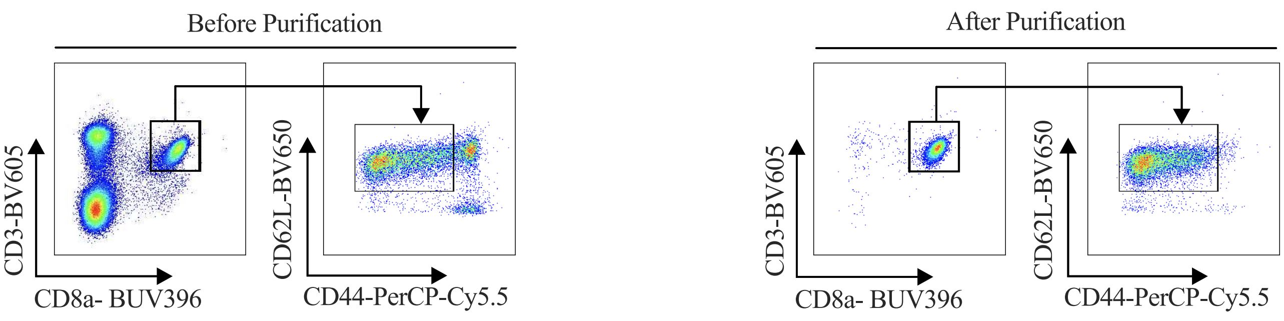

Supplementary Figure 2

a Original Western blot [Fig.2 d]

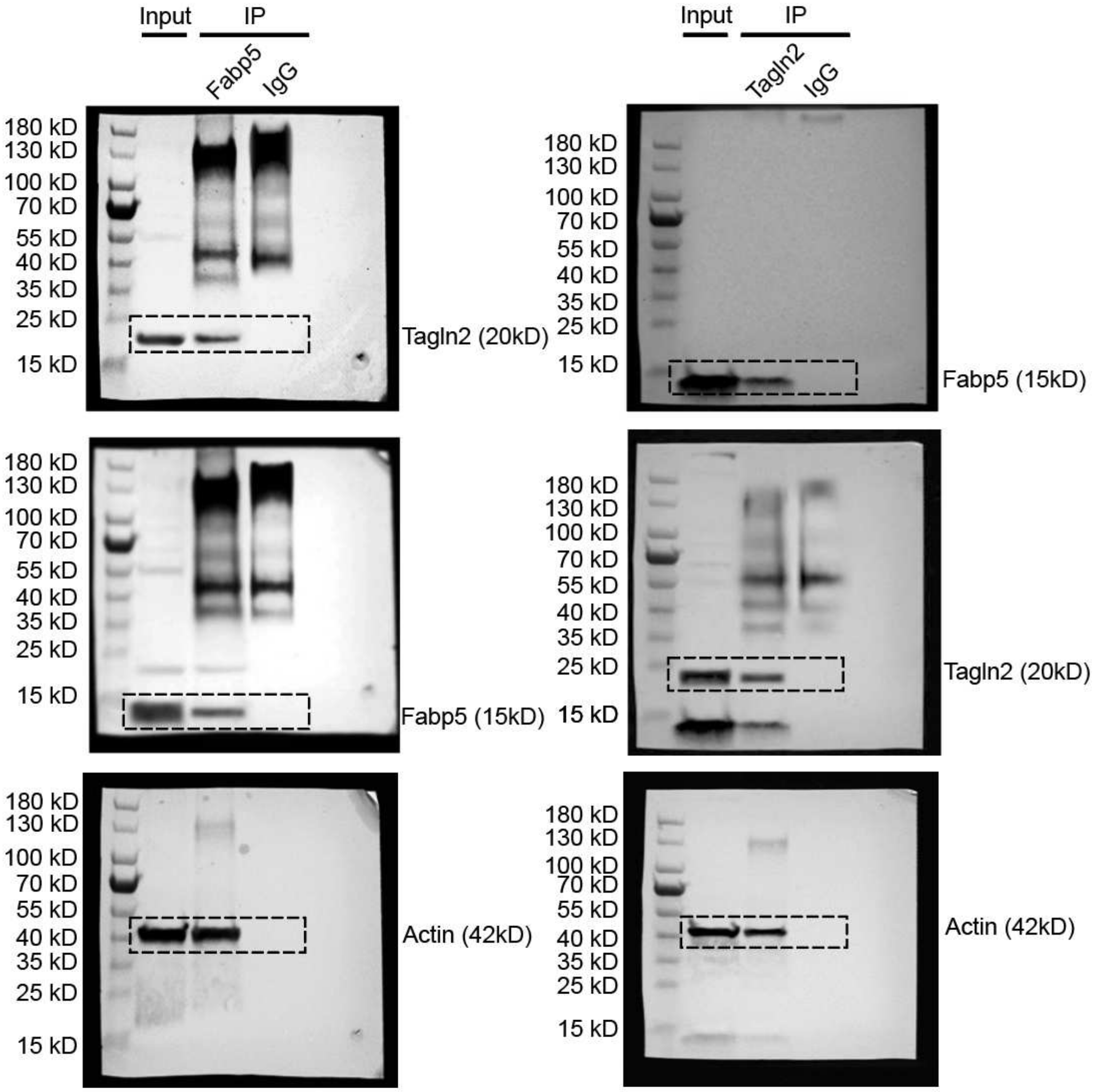

***Note: Dotted squares identify cropped areas**

b Original Western blot [Extended Data Fig. 7c]

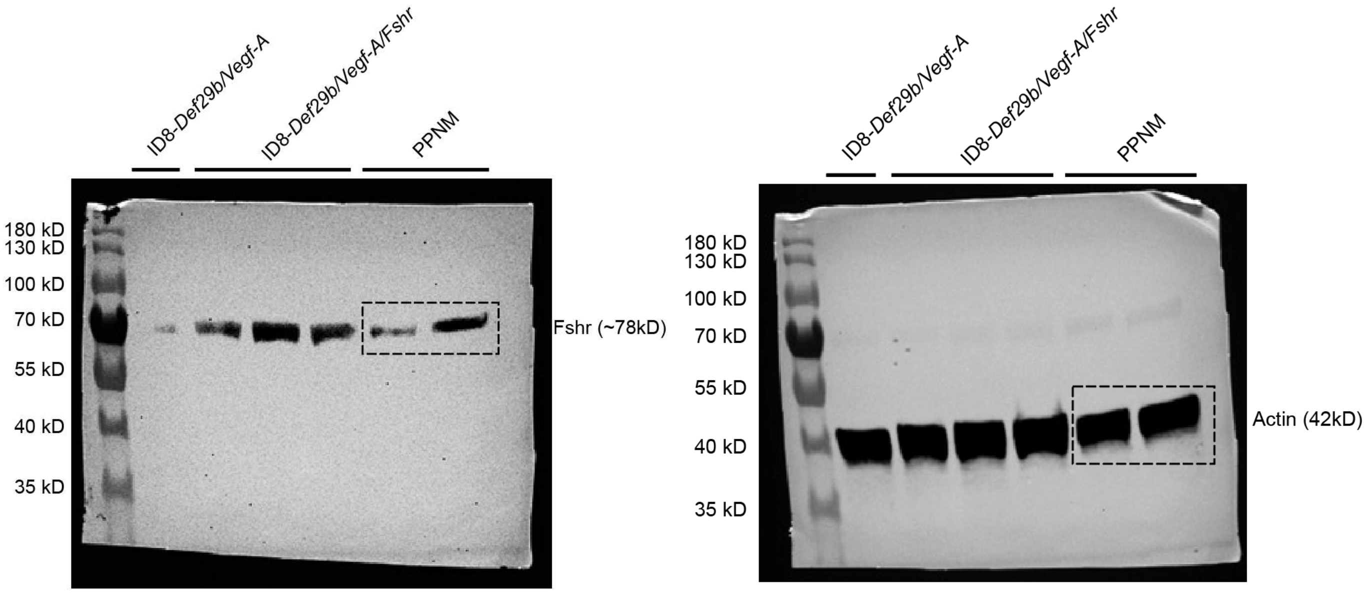

***Note: Dotted squares identify cropped areas**
